# LARa: Creating a Dataset for Human Activity Recognition in Logistics Using Semantic Attributes

**DOI:** 10.3390/s20154083

**Published:** 2020-07-22

**Authors:** Friedrich Niemann, Christopher Reining, Fernando Moya Rueda, Nilah Ravi Nair, Janine Anika Steffens, Gernot A. Fink, Michael ten Hompel

**Affiliations:** 1 Chair of Materials Handling and Warehousing, TU Dortmund University, Joseph-von-Fraunhofer-Str. 2-4, 44227 Dortmund, Germany; christopher.reining@tu-dortmund.de (C.R.); nilah.nair@tu-dortmund.de (N.R.N.); janine.steffens@tu-dortmund.de (J.A.S.); michael.tenhompel@tu-dortmund.de (M.t.T.); 2 Pattern Recognition in Embedded Systems Groups, TU Dortmund University, Otto-Hahn-Str. 16, 44227 Dortmund, Germany; fernando.moya@tu-dortmund.de (F.M.R.); gernot.fink@tu-dortmund.de (G.A.F.)

**Keywords:** human activity recognition, attribute-based representation, dataset, motion capturing, inertial measurement unit, logistics

## Abstract

Optimizations in logistics require recognition and analysis of human activities. The potential of sensor-based human activity recognition (HAR) in logistics is not yet well explored. Despite a significant increase in HAR datasets in the past twenty years, no available dataset depicts activities in logistics. This contribution presents the first freely accessible logistics-dataset. In the ’Innovationlab Hybrid Services in Logistics’ at TU Dortmund University, two picking and one packing scenarios were recreated. Fourteen subjects were recorded individually when performing warehousing activities using Optical marker-based Motion Capture (OMoCap), inertial measurement units (IMUs), and an RGB camera. A total of 758 min of recordings were labeled by 12 annotators in 474 person-h. All the given data have been labeled and categorized into 8 activity classes and 19 binary coarse-semantic descriptions, also called attributes. The dataset is deployed for solving HAR using deep networks.

## 1. Introduction

Human activity recognition (HAR) assigns human action labels to signals of movements. Signals are time series that are obtained from video-frames, marked-based motion capturing systems (Mocap), or inertial measurements. This work focuses on HAR using Mocap and inertial measurements. Methods of HAR are critical for many applications, e.g., medical and rehabilitation support, smart-homes, sports, and in industry [1,2,3]. Nevertheless, HAR is a complicated task due to the large intra- and inter-class variability of human actions [1]. In addition, extensive annotated-data for HAR is scarce. This is, mainly, due to the complexity of the annotation process. Moreover, datasets of HAR are likely to be unbalanced. Usually, there exists more samples of frequent activities, e.g., walking or standing in comparison with picking an article [4,5].

Warehousing is an essential element of every supply chain. The main purpose of warehousing is storing articles and satisfying customers’ orders in a cost and time-efficient manner. Despite an increase in automation and digitization in warehousing and the impression of a shrinking number of employees, the employee numbers are rising [6,7]. Manual order-picking and -packing are labor-intensive and costly processes in logistics. Information on the occurrence, duration, and properties of relevant human activities is fundamental to assess how to enhance employee performance in logistics. In the state-of-the-art, manual activities of the employees are mostly analyzed manually or analytically, using methods such as REFA Time Study [8] or Methods-Time Measurement (MTM) [9].

The potentials of sensor-based human activity recognition (HAR) in logistics are not yet well explored. According to Reining et al. [10], in the past ten years only three publications dealt with HAR in logistics [11,12,13]. One major reason for this is the lack of freely accessible and usable datasets that contain industrial work-processes. This is because industrial environments such as factories and warehouses pose a challenge for data recording. Regulations such as the European General Data Protection Regulation [14] further create barriers when handling sensitive data, such as the work performance of employees and their physical characteristics. Thus, scientists tend to fall back to pseudo-industrial laboratory set-ups for dataset creation. The closeness to reality of these low-scale laboratory set-ups is often questionable. For example, the recognition performance in [10] suffered from the recording procedure that split a workflow into activities that were each recorded individually. This was the case because the transitions between activities were not examined properly.

In the ’Innovationlab Hybrid Services in Logistics’ at TU Dortmund University, manual processes of a real-world warehouse are replicated on an area of 220 m${}^{2}$ [15]. Fourteen individuals carry out picking and packaging activities in three scenarios under real-life conditions. All activities are recorded in a sensor-rich environment, using an Optical marker-based Motion Capture (OMoCap) system, three sets of inertial measurement units (IMU), and an RGB camera. All data streams are synchronized. In total, **LARa** contains 758 min of data. Twelve annotators labeled the OMoCap data in 474 person-h (PHR). A subsequent revision took 143 PHR for 4 revisers. Data are labeled using 8 activity classes and 20 binary coarse-semantic descriptions. These descriptions will be denoted as attributes [16].

Traditional methods of statistical pattern recognition have been used for HAR. These methods segment signal-sequences using a sliding-window approach, extract relevant features from the segmented sequences and train a classifier for assigning certain action labels. Recently, deep architectures have been used successfully for solving HAR problems. They are end-to-end architectures, which are composed of feature extractors and a classifier. They combine learnable convolutional operations with non-linear functions, downsampling operations, and classification layer [2,3,17,18]. These architectures map sequence segments of measurements from multichannel sensors into a single class or a semantic-based representation [16]. Stacked convolution and downsampling operations extract abstract and complex temporal relations from these input sequences.

Attribute-based representations have been deployed for solving HAR. Attributes describe activity classes semantically [16]. For example, *handling* can be represented by moving the *left*, *right*, or *both* hands, and by its *pose* based on a picked article. *Right-hand*, *left-hand* and *box* can be considered as attributes. Attributes are used for sharing high-level concepts among activity classes. They are an additional mapping between sequence measurements of the data streams and activity classes. In [13,16], a single combination of attributes represents an activity class. Nevertheless, this limits the properties of attribute representations. As human actions vary, they could be represented by different combinations of attributes.

This paper introduces a novel and large dataset for HAR in the context of logistics. This dataset contains annotations of human actions and their attributes in the intra-logistics. This paper explains in detail the recording scenarios, sensors settings, and the annotation process. In addition, it presents the performance of employing deep architectures for solving HAR on the provided dataset. It describes an approach for adapting deep architectures to solve HAR using attribute representations. For the dataset, the detailed annotation of these attributes leads to a total of 204 unique attribute representations for the 8 activity classes. This high level of granularity is the prerequisite for evaluating different activity recognition approaches. The **LARa** dataset contains labeled IMU and OMoCap data, the respective RGB videos, the recording protocol as well as the annotation and revision tool. All data are freely accessible: https://doi.org/10.5281/zenodo.3862782.

The contribution answers also the following research questions in the context of the first freely accessible logistics HAR dataset—**L**ogistic **A**ctivity **R**ecognition Ch**a**llenge (**LARa**):

1. What is the the state-of-the-art of dataset creation for multichannel time-series HAR?
2. What guidelines are proposed for creating a novel dataset for HAR?
3. What are the properties of a logistics-dataset for HAR created by following these guidelines?
4. How does a tCNN perform on this dataset using softmax compared to an attribute representation?

This contribution is organized as follows. Section 2 presents the related work on multichannel-time series HAR. In Section 3, the freely accessible dataset **LARa** is introduced. First, data recording steps in the logistics scenarios are presented. Second, the activity classes and semantic attributes are explained. Third, findings of the annotation and revision process are highlighted. Section 3 concludes with an overview of the **LARa** dataset. Section 4 presents an example of solving HAR on the **LARa** dataset using deep architectures. Finally, Section 5 offers a discussion and the conclusions of the work in this contribution. Additionally, Appendix A gives an overview of state-of-the-art datasets for HAR. Based on the datasets’ descriptions, the guideline for creating the novel dataset in Section 3 is derived.

## 2. Related Work

Methods of supervised-statistical pattern-recognition have been used successfully for HAR. The standard pipeline consists of preprocessing, segmentation, statistical-features extraction, and classification. High and low-pass filters are common as preprocessing steps. High-pass filters serve denoising, as faulty measurements in the sensors are on the high-frequency spectrum. In addition, changes in human motions are rather in the low frequency. Low-filter operations are used for separating gravitation and inclination of the IMUs in constant space, i.e., the earth [19]. A segmentation approach, e.g., a sliding window, divides the input signal into segments of a certain time duration. Statistical features are computed from the time and frequency domain. They are, for example, the mean, variance, channels-correlation, entropy, energy, and coherence. [1,11,20,21]. The authors in [10] present a summary of such features. Using these features, the parameters of a classifier are computed. The classifier assigns an activity label to an unknown input. Some examples of classifiers are Naïve Bayes, Support Vector Machines (SVMs), Random Forests, Dynamic Time Warping (DTW), and Hidden Markov Models (HMMs) [11,22]. These methods, however, might show low performance on challenging HAR problems. In addition, different combinations of features must be selected manually per activity. This makes the method hardly scalable and is prone to overfitting [3,19].

The authors in [11] evaluate HAR for the order picking using statistical pattern recognition. They present a novel dataset of human order picking activities. They use a low number of sensor devices. Specifically, they deployed three inertial measurement units (IMUs), which are worn by workers in two different scenarios. They computed handcrafted-statistical features on segments that were extracted from the sliding window approach. The authors evaluated three classifiers, namely, an SVM, a Naïve Bayes, and a Random Forest. The authors in [19] solve HAR for activities on daily living. They compute statistical features on three data streams, namely the raw inertial-measurements, their AC and DC components. They propose a hierarchical approach with bagging performance of simple classifiers on a different combination of device locations on the human body.

Deep architectures have been also deployed for solving HAR. Temporal Convolutional Neural Networks (tCNN), Recurrent Networks (RNN), e.g., Long Short-Term Memory (LSTMs), and a combination of both are examples of architectures in the field. tCNNs are hierarchical architectures that combine the feature extraction along with time and classification in an end-to-end approach. They learn the features and parameters of the classifier directly from raw data. tCNNs are presented in [17,18,23]. They are composed of convolution and pooling operations that are carried out along the time axis. tCNNs exploit their hierarchical composition becoming more discriminative concerning human actions. The combination of stacked convolutional and pooling layers find temporal relations that are invariant to temporal translation. They are also robust against noise. Moreover, these architectures share small temporal filters among all the sensors in the IMUs. Local temporal-neighborhoods are likely to be correlated independent of the sensors’ type. The authors in [2] introduce an architecture that combines temporal convolutions and LSTMs layers replacing the fully-connected layers. LSTMs are recurrent units with memory cells and a gating system, which are suitable for learning long-temporal dependencies in sequences. These units do not suffer from exploiting or vanishing gradients during training. The authors in [24] utilize a shallow recurrent network; namely, a three-layered LSTM and a one-layered bidirectional LSTM. Bidirectional LSTMs process sequences following their inputs in both forward and backward directions. The performance of the BLSTMs outperforms the convolutional architectures. Nevertheless, tCNNs show more robust behavior against parameter changes. The authors in [3] propose a tCNN that is adapted for IMUs, called IMU-CNN. The architecture is composed of convolutional branches corresponding to each IMU. These branches compute an intermediate representation per IMU. They are then combined in the last fully-connected layers. The authors compared IMU-CNN with the tCNN and a tCNN-LSTM, similar to [2]. The IMU-CNN shows a better performance, as it is more robust against IMU’s faults and asynchronous data. The authors in [20] investigate the effect of data normalization on the deep architecture’s performance. They compare the normalization to zero-mean and unit standard deviation, batch normalization, and a pressure-mean subtraction. The architecture’s performance improves when utilizing normalization techniques. Extending the work of [3], the authors use four sensor fusion strategies. They find that late fusion strategies are beneficial. Additionally, they evaluate the robustness of the architectures concerning proportions of the training dataset.

The authors in [16] propose using attribute-based representation for HAR. In object recognition and word-spotting problems, attributes are semantic descriptions of objects or words. They represent coarsely a class. In [12], a search for attributes is presented, as there are no datasets with such annotations. The selected attributes are better suited for solving HAR. For such a search, the authors deploy an evolutionary algorithm. Firstly, they assign random binary representations to action classes as population. Secondly, they evaluate a population using deep architectures with a sigmoid activation function. The validation’s performance serves as evolution fitness. The authors deploy non-local mutations on the populations. They conclude that using attribute representations boosts the performance of HAR. Even, a random attribute-representation performs comparably to a directly classifying human actions. A drawback of this approach was the lack of a semantic definition of the attributes.

Attribute-based representations have been deeply explored on HAR in [13]. Particularly, in the manual order picking process, attribute representations were expected to be beneficial for dealing with the versatility of activities. This contribution compared the performance of deep architectures trained using different attribute representations, and it evaluated their quantitative performance as well as their quality from the perspective of practical application. Expert-given attribute representations performed better than a random one, created following the conclusions in [16]. A semantic relation between attributes and activities enhances HAR not only quantitatively with regards to performance, but it also ensures a transfer of the attributes between activities by domain experts. In this preliminary work, the mapping between activity classes and attribute representations was one-to-one. This became a multiclass problem that limits the benefits of attribute-based representations.

An important element of these supervised methods is annotated data [20]. A drawback of using deep methods is the need for extensive annotated data. This contrasts against the statistical pattern recognition. However, capturing and annotating data for HAR is laborious and expensive. Moreover, annotations regarding attributes are not existing. These fine-grained annotations represent an extra cost. In [13], human actions were given unique attribute representations. Nevertheless, human actions might include a different combination of attributes. Different combinations of attributes might be helpful for zero-shot learning and reducing the effects of the unbalanced problem. They also might allow clustering signals of a certain activity but with slight changes in the human movements. So far, there is no large-scale, freely accessible dataset of human activities in complex, industrial processes; neither using attributes. In addition, there are not standard guidelines for creating such a dataset. Thus, it needs to be defined beforehand. A review of existing datasets and their shortcomings in regards to the goal of this paper is presented in the appendix to further motivate the introduction of the new dataset in the following section.

## 3. Introducing the LARa Dataset

This section states the **LARa** dataset’s specifications. Requirements and specifications of **LARa** are based on a detailed review of datasets for HAR, see Appendix A. In particular, the origin of the laboratory set-ups, the subjects’ characteristics as well as the recording and annotation procedure are showcased. For data recording, the researchers created physical replicas of real-world warehouses in a laboratory. They are called scenarios in this contribution. This subsection gives insights into the replicas’ creation, and it explains the underlying warehousing processes. Next, the sensors’ configuration and the proper preparation of the subjects are presented.

### 3.1. Guidelines for Creating and Publishing a Dataset

The datasets, as discussed in the Appendix A, show no uniform guidelines for dataset creation. Based on this overview of the datasets and their description, a guideline for the creation of a dataset is derived.

If possible, the recording should take place under real conditions. Realistic environments ensure recording natural movements, e.g., a real warehouse or a detailed replica. A replica requires a large laboratory. In addition, objects similar to the real scenarios are needed, e.g., a picking card. The subjects’ selection depends on the variety of people from the real environment, e.g., employees of a real warehouse. The selection terms involve age, sex, height, and handiness.

In addition to the realistic environment, the behavior of the subjects should be implemented as naturally as possible. Instead of just recording individual activities in isolation, recording a whole process enables natural behavior and thus natural movements. A recording should therefore not only consist of one activity, e.g., lifting a box, but should occur as part of a process, e.g., lift the box→walk with the box→pick the article→put the article in the box→walk with the box→place the box. Using a recording protocol and RGB camera for documentation, discrepancies, such as the slipping of sensors or markers, are noticeable after the recordings.

It is recommended to use different sensor types with a high frame rate. Since there is no uniform positioning of sensors, several sets of different positions on the human body can be experimented with.

OMoCap and RGB videos could help in complex annotation-scenarios. The annotation is to be carried out by domain experts such as physiotherapists, dance teachers, or, in the case of logistics, logistics experts. As soon as several people annotate or are expected to benefit from the annotated data, an annotation guideline is necessary. A revision of the annotation is recommended to improve the quality of labeled data. To ensure other applications, the representation of the activity classes should be as granular as possible. The granularity depends on the number of activities and can be increased by a binary coarse-semantic description.

Necessary general information such as location and period of the recordings must be specified. The method of data acquisition and the description of the activities are part of the description of the dataset. In addition to the method of annotation and its effort, the labeled activity classes must be described. The dataset should contain labeled and raw data from all sensors. Access to the annotation tool must be guaranteed for understanding the process of annotation.

### 3.2. Laboratory Set-Ups based on Logistics Scenarios

This subsection explains three logistics scenarios for data recording. The warehousing processes’ graphical representation is based on the guidelines defined by the Object Management Group [25]. The graphical and textual descriptions of the scenario guide researchers when applying methods of HAR that take context into consideration. A detailed explanation of the scenarios might be helpful for approaches involving context, preconditions and effects, e.g., Computational Causal Behavior Models (CCBM) [26]. This context may be the constraints of the warehousing process. For example, some activities can only be performed in a specific order or at a specific location and time.

Data were recorded in physical set-ups created in a controlled environment—the ’Innovationlab Hybrid Services in Logistics’ at TU Dortmund University [15]. A group of researchers created the physical replica of warehousing scenarios following a cardboard engineering approach [27,28].

#### 3.2.1. Logistics Scenario 1—Simplified Order Picking System

**Figure 1:**
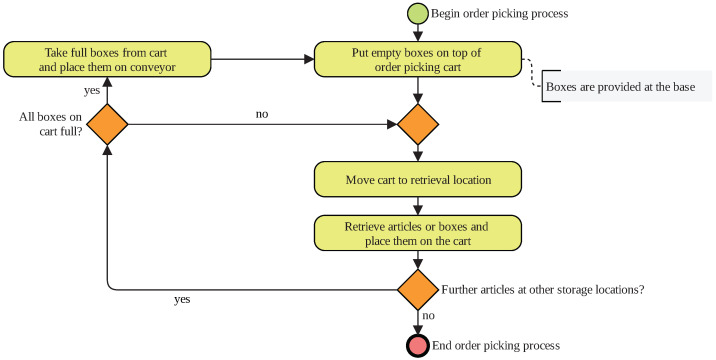
Business process model of logistics Scenario 1—simplified order picking.

**Figure 2:**
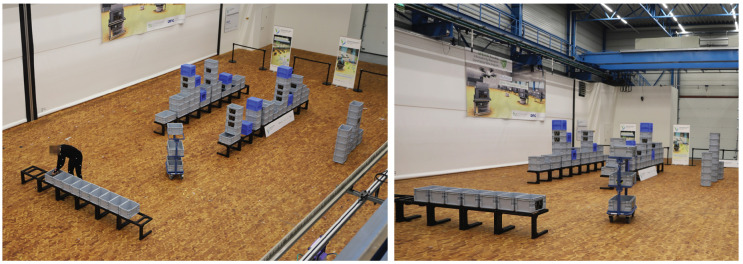
Physical laboratory set-up of logistics Scenario 1—simplified order picking.

The first scenario is not based on a real warehouse. Nevertheless, this process may exist in reality. The process is illustrated in Figure 1, the physical laboratory set-up is presented in Figure 2.

In the beginning of an order-picking process, the subject places boxes on an empty order-picking cart. These empty boxes are provided at the base. In a real warehouse, this base may be a conveyor that transports empty boxes to the order picker while transporting full boxes to the shipping area. In the laboratory, stacking frames recreated the conveyor. This simplification does not influence human-motion behavior. The boxes and the cart were standard items that are common in the industry.

Next, the subject moves the cart to a retrieval location. The researchers who guided the recordings specify where to go. An order-picking aisle was recreated by placing boxes on frames. When the subject arrives at a retrieval location, they pick articles from a box or they open a fronted bin. The subjects place the articles in an empty box on their cart. The articles were small, light items, such as bags of 500 g. This procedure of taking the cart to a new location and retrieving goods is repeated until all boxes on the cart are full. The subject takes the cart back to the base and places the full boxes on the conveyor. The order-picking process starts anew. When all articles in the aisles’ boxes are empty, the order-picking process has to end. The research team refills the boxes.

#### 3.2.2. Logistics Scenario 2—Real-World Order Picking and Consolidation System

**Figure 3:**
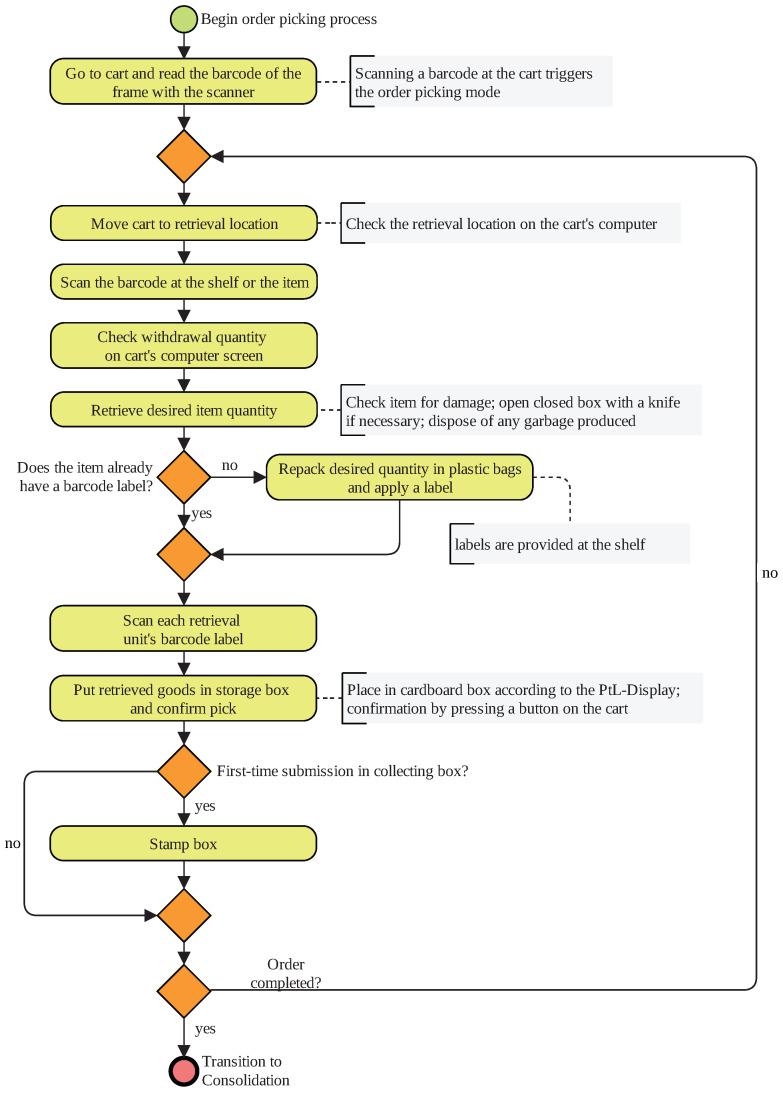
Business process model of logistics Scenario 2 (Part 1)—real warehouse order picking.

**Figure 4:**
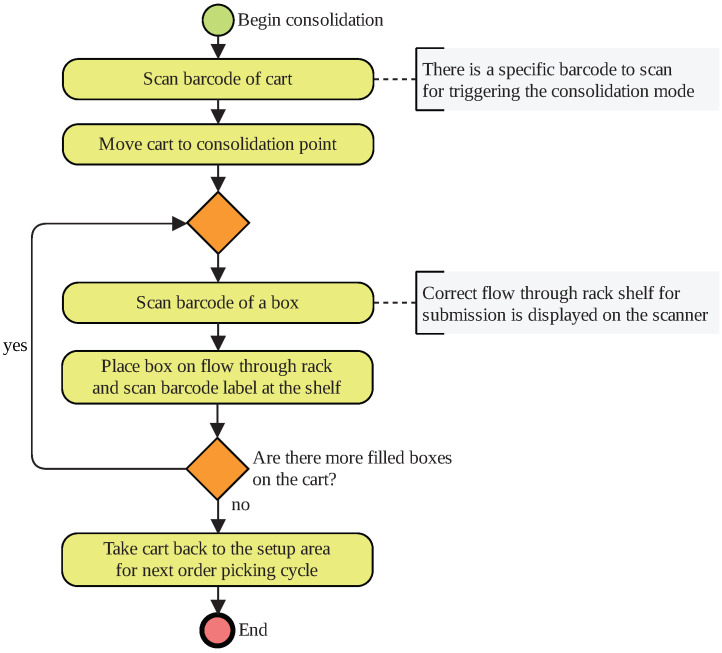
Business process model of logistics Scenario 2 (Part 2)—real warehouse order picking.

**Figure 5:**
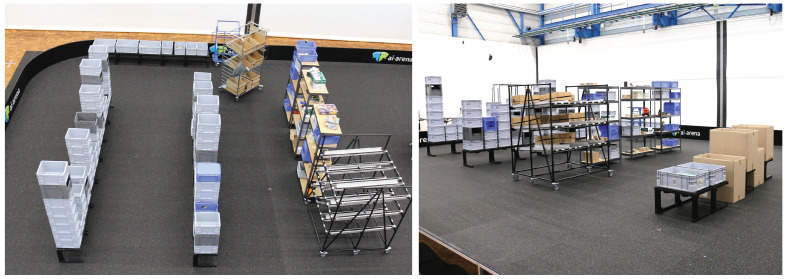
Physical laboratory set-up of logistics Scenario 2—real warehouse order picking.

The second scenario is based on a real warehouse. Access to the site and process documentation was granted by industry partners of the chair of Materials Handling and Warehousing. In contrast to Scenario 1, the second scenario takes information technology processes such as scanning barcode labels or pushing buttons for pick confirmation into account. For the sake of clarity, the order-picking process and the consolidation process of the picked goods are illustrated separately in Figure 3 and Figure 4, respectively. The physical laboratory set-up of Scenario 2 is illustrated in Figure 5.

The order-picking cart is bigger than the one used in the first scenario as visible in Figure 2 and Figure 3. It has three shelves of equal size that are filled with cardboard boxes of different shapes and sizes. Each box is held open with a rubber band. In the real warehouse, a so-called put-to-light (PtL) frame is attached to the cart. It gives a visual signal where to place articles and has buttons to press for retrieval and submission confirmation. Small calculators are attached to the cart to replicate this system in the laboratory. On its shorter end, the cart has two handles, a small screen, a stamp pad, a plastic bag for packaging waste and a second bag, which is filled with more small plastic bags. Apart from the screens, all items could be purchased. A labeled cardboard replicates the screen. The research group gives information to the subject, which is usually displayed on the screens. For example, this information might be the retrieval location or the picking quantity.

Subjects deploy a stamp and a knife. They are attached to the OMoCap suit. Additionally, subjects operate a handheld scanner, which is attachable to the cart. To assure a natural motion of the subjects when using the scanner, all items have barcode labels that need to be scanned. Thus, the subjects have to use the scanner correctly to trigger an acoustic signal that confirms a scan operation.

An order consists of several items that need to be picked in varying quantities. For each order-picking cycle, one cart works on the orders of several customers at the same time. This is referred to as order batching. The articles are household goods of varying dimensions and weights, such as cutlery, dishes, or storage boxes. They are stored in plastic and cardboard boxes and open lid bins. Some of the cardboard boxes were sealed with tape for protecting the goods. These storage units are placed on shelves with different heights or on the ground. Stacking frames and shelves formed two aisles. In the real-world system, a flow-through rack is deployed for goods consolidation. In the laboratory, pipe-racking systems were used to recreate it. Each chute of the flow-through rack is equipped with a barcode label and a human-readable ID.

In general, the subject scans all labeled units to ensure that the correct article is picked, e.g., a single article or a newly labeled plastic bag. There are three cases for scanning an article’s barcode label. In the first case, the articles are individually packed. Every article already has a barcode label attached. Second, some articles are in a secondary packing, e.g., a cardboard box or a plastic bag that needs to be opened before retrieval. The articles in this secondary packaging have an individual barcode. Third, some articles do not have an attached-barcode label. In this case, the barcode at the shelf has to be scanned. There is a barcode label roll, which is provided next to the respective articles. These labels need to be attached to the retrieval unit.

To begin the order-picking process, the subjects scan the barcode of the cart to trigger the order-picking mode. The screen shows the next retrieval location. When they arrive there, they scan the article’s barcode label, which may be found on the article, or on the shelve as explained previously. If the article is correct, the screen indicates the correct withdrawal quantity.

Next, the subject retrieves the correct amount of articles. If necessary, they open sealed cardboard boxes with the knife. They dispose packaging waste using the plastic bag at the cart. If the article already has a barcode label, the subject can scan it so that the PtL-Frame visually indicates the correct box to submit the articles. For articles that do not have a barcode label, the subject wraps the desired quantity of articles in a plastic bag and seals it with a barcode label provided at the shelf.

Pressing a button confirms each submission into a box on the cart. The button is on the PtL-frame above the box. If this is the first item in a box, the box must be marked with a stamp. This is a quality assurance to trace back the employee who packed the box. The subject takes the cart from one retrieval location to the next until the order is complete.

The order-picking process is proceeded by the consolidation of the packed goods for dispatching preparation. For consolidation, the boxes must be inserted on the back side of a flow-through rack. On the front site the packaging, workplaces are located where dispatch preparation takes place. As with the order-picking mode, the subjects scans a specific barcode on the code to trigger the consolidation mode. Next, they take the cart to the consolidation point, which is shown on the cart’s display. The subjects scan the barcode of a box so that the scanner’s display shows the correct chute. After they inserted the box, they scan the barcode label at the chute to confirm the submission. This procedure repeats until there are no more boxes on the cart.

#### 3.2.3. Logistics Scenario 3—Real-World Packaging Process

**Figure 6:**
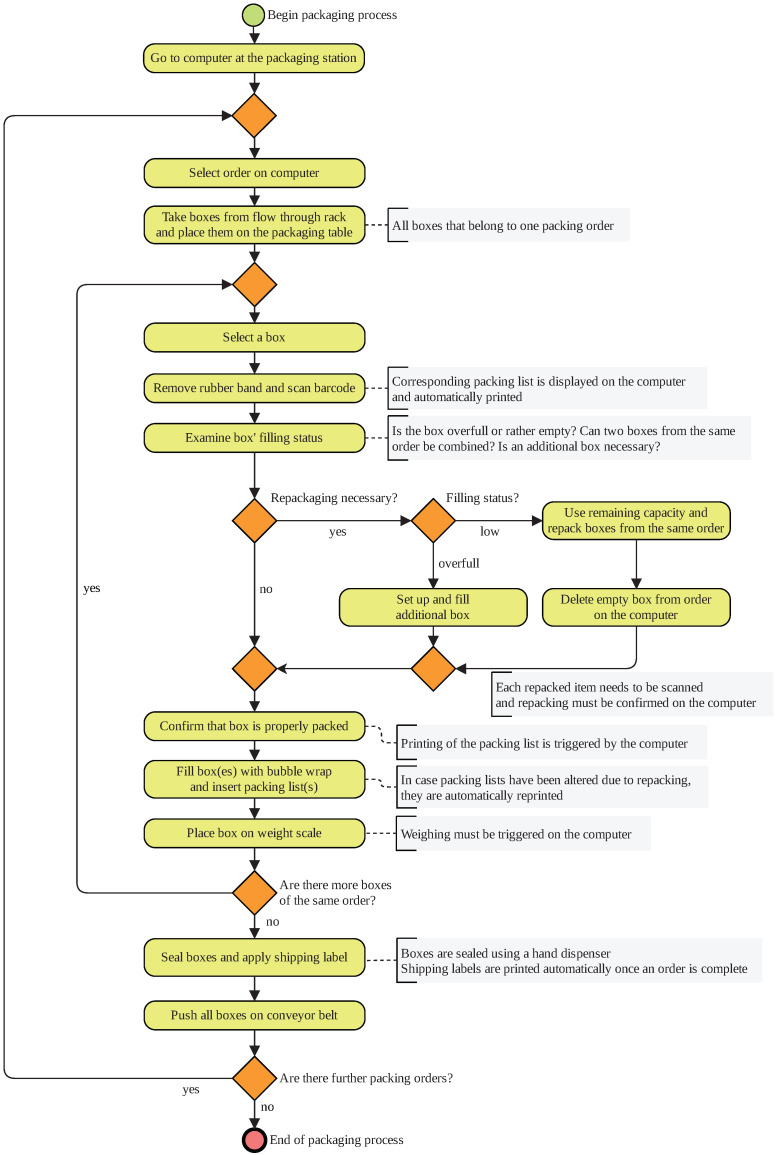
Business process model of logistics Scenario 3—real warehouse packaging work station.

**Figure 7:**
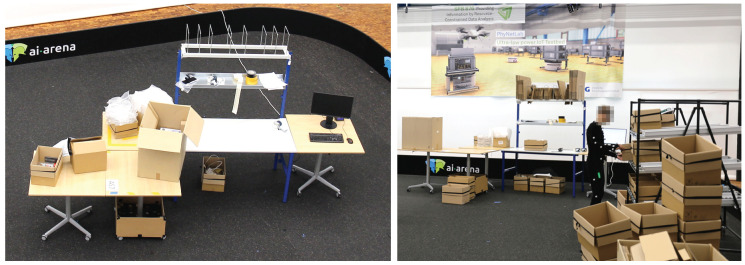
Physical laboratory set-up of logistics Scenario 3—real warehouse packaging work station.

The third scenario is the packaging process that follows the order picking and consolidation of scenario 2 in the same real-world warehouse. The packaging process serves the dispatch preparation of the picked articles. In general, the consignment size per order does not exceed 5 boxes. Thus, the shipping by pallet is not feasible. The real-world packaging process is illustrated in Figure 6. Its physical laboratory set-up can be observed in Figure 7.

Each packaging workplace is equipped with a computer, a printer, a bubble wrap dispenser, a tape dispenser, a scale for weighing boxes, and a trash bin. Next to the table, a conveyor is located where all boxes have to be placed that are ready for shipment. The packaging table in the laboratory is a model often found in real warehouses. Further tables were placed next to the packaging table to provide space for the equipment. The table on the far left was used to recreate the surface of the conveyor. When a box was pushed onto the surface, a researcher took the box. The actual motion of a conveyor is not necessary to ensure a human motion that is close to reality. The dimensions of the tables in the laboratory closely resemble the table from the real-world warehouse.

For the tools, equipment has been purchased that is similar to the real-world system. The bubble wrap dispenser was recreated by cutting a small opening in a cardboard box. The wrap was refilled manually by the researchers present during the recordings. A fully functional computer was placed on the table. Mouse and keyboard were attached to the computer and a spreadsheet application was running on it. When computer work was necessary, the subjects were tasked to perform basic tasks in the program. The printers were substituted by a researcher handing the printed items to the subject. As the weight scale is an area on the table’s surface, it could be recreated by indicating a certain area with colored stripes.

As explained previously, all boxes to be prepared for shipment were stored in a flow-through rack. During recordings, the rack from Scenario 2 was used. It was moved next to the packaging table. When recordings were conducted, second and third scenarios were in immediate succession, the flow-through rack was already filled with boxes, which were filled with articles.

By the beginning of the packaging process, the subject goes to the computer and chooses a packing order. Next, they take all boxes that belong to one order from the flow-through rack and place them on the packing table. The rubber band of each box is removed and the barcode needs to be scanned with the hand scanner. When doing so, the packing list of the order is printed automatically.

For each box, the subject evaluates its filling level to decide whether repacking is necessary. This is the case when the box is either rather empty or overfull. In the first case, more articles from a different box of the same order are added. In the second case, the articles protrude the box. Articles may be bigger than the box, due to incorrect article master-data. When the filling level is low, the contents of several boxes are combined. When a box is removed from the order, this information must be entered into the computer. Contents of an overfilled box are put into a bigger one. The subject can get boxes of different sizes from storage next to the packing table. When repacking articles from one box to another, each one needs to be scanned and the repacking must be confirmed at the computer.

The subject confirms that all boxes of an order are filled properly. In case the packing list has been altered due to repacking, it is reprinted automatically. Next, the subject puts the packing list in each box and fills them up with bubble wrap. Then, each box must be pushed onto a scale. The subjects need to trigger the weighing process at the computer. The system will check if the actual weight of the box corresponds to the expected weight according to the master data and the packing list.

Once all boxes are packed correctly and their weight has been approved, the subjects seal them using a tape dispenser. The printer automatically prints the shipping labels when all boxes of one order are ready to be sealed. The subject applies a label to each box. Eventually, each box is pushed onto the conveyor surface.

### 3.3. Configuration of Sensors and Markers

**Figure 8:**
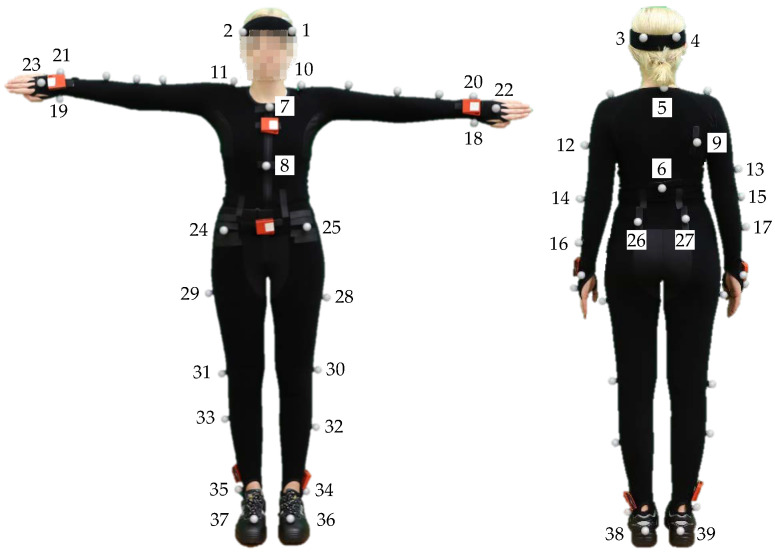
Marker position on a Optical marker-based Motion Capture (OMoCap) suit.

**Figure 9:**
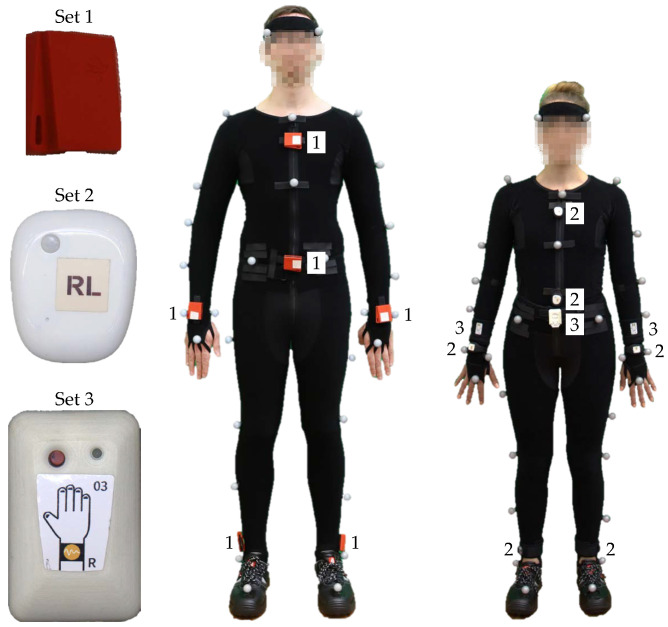
Positions of on-body devices (inertial measurement unit (IMU)) from set 1 (Texas Instruments Incorporated), set 2 (MbientLab), and set 3 (MotionMiners GmbH).

The OMoCap system tracked 39 reflective markers from a suit, see Figure 8. A VICON system consisted of 38 infrared cameras recording at a sampling rate of 200 fps. Three different sets of on-body devices or IMUs record tri-axial linear and angular acceleration, see Figure 9. IMU-sets 1 and 3 served as proof of concept and they are not part of the dataset. The six IMUs of the second set from MbientLab [29] are attached to the arms, legs, chest, and waist. They record tri-axial linear and angular acceleration at a rate of 100 Hz.

### 3.4. Characteristics of Participating Subjects

A total of 14 subjects (S) were involved in the recording process. Their characteristics, including sex, age, weight, height, and handedness, are listed in Table 1. Examining the minimum and maximum of these characteristics show that a wide spectrum of physical characteristics is present. Thus, the subjects’ motion patterns vary widely. In addition, the ratio of left-handed to right subjects closely resembles the general population [30,31].

**Figure 10:**
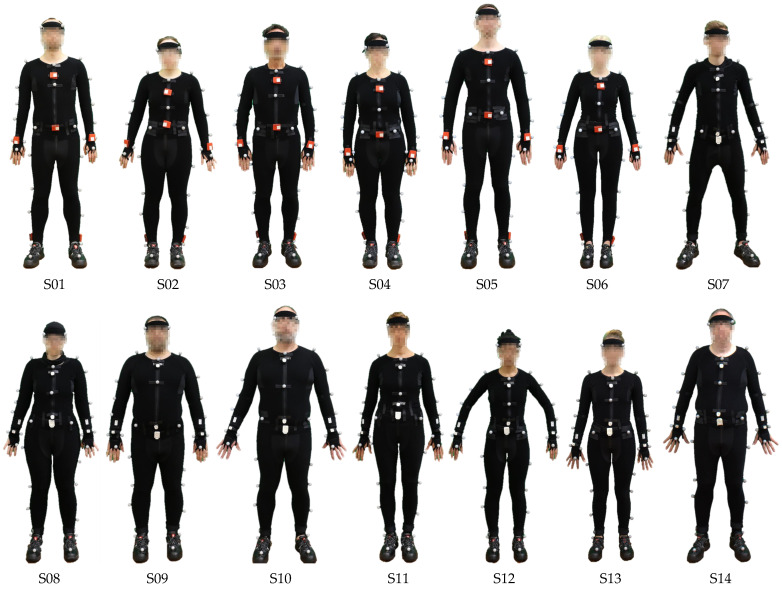
Subjects before the recordings.

All subjects participated in a total of 30 recordings of 2 min each, which corresponds to about 30 recording/subject × 2 min/recording × 14 subject = 840 min of recorded material. In Scenario 1, subjects 1 to 6 performed 30 recordings wearing the OMoCap suit and the IMU-set 1. Subjects 7 to 14, wearing the OMoCap suit and the IMU-sets 2 and 3, participated in 2 recordings in Scenario 1, 14 recordings in Scenario 2 and 14 packing recordings in Scenario 3. Due to heavy noise and issues with the sensor readings, some recordings had to be scrapped, and they are not included in the dataset. Thus, the number of recordings per subject deviates in Table 1. A total of 379 recordings (758 min) were annotated and are included in the dataset. Figure 10 shows the varying physical features of all subjects true-to-scale.

### 3.5. Recording Procedure

The **LARa** dataset was recorded in 7 sessions. In the first 3 sessions, subjects 1 to 6 went through Scenario 1. In sessions 4 to 7, data were recorded in all three scenarios with subjects 7 to 14.

#### 3.5.1. Preliminaries

Before the recording, each subject was measured according to the information necessary for the VICON Nexus software: body mass, height, leg length, knee width, ankle width, shoulder offset, elbow width, wrist width, and hand thickness. Subsequently, the test subjects were equipped with an OMoCap suit, a headband, and work safety shoes, as used in real warehouses. Markers and IMUs were attached to the suit. To document the proper positioning of all markers and IMUs, each subject was photographed from four sides before the recording.

#### 3.5.2. Recording Process

For the sake of recording realistic motions, the subjects were introduced to the scenarios by a domain expert in advance. Test runs were carried out before recordings commenced. The subjects were allowed to familiarize themselves with the processes and objects. The subjects do not perform individual and isolated movements as in other datasets that originate from laboratories, e.g., [32]. Rather, realistic motion sequences were the goal. To achieve this, the subjects were only instructed about their tasks within a scenario. They were not told how to perform specific motions necessary to fulfill their task. Thus, the way they handled items, picked boxes and moved to a location were not influenced by the researchers. The motion is solely determined by each subject’s individual preference. In addition, the subjects were not given detailed information about the underlying research goal to avoid a bias in their motion behavior.

Between each recording unit of two min, a break of only a few seconds was necessary to start the next capture. Hence, the subjects would be able to remain focused on the task. The subjects did not take off the suit between recordings. After the recordings concluded, each subject was photographed again from four sides to reassure the proper positioning of the markers and sensors.

#### 3.5.3. Documentation and Protocol

A protocol was kept before, during and after the recordings for each subject to ensure repeatability of the recording sessions: time, size of the suit and shoes, room temperature, the use of velcro to fit the suit to the person, RGB video files that were created, number and descriptions of photos taken, remarks, and incidents.

The expenditure of time for recording is made up of the OMoCap system’s calibration, the preparation of each subject, their introduction to the scenarios and the recordings. In total, the expenditure was over 197 PHR (8.22 days) to record 14 h of material. To support the subsequent annotation of the data, the sessions were captured by a RGB camera. In sessions 1 to 3, only occasional recordings were created with a RGB camera, but at least one recording per subject is available. Due to the increasing complexity of human motion and the increasing spectrum of objects in Scenarios 2 and 3, i.e., the subjects 7 to 14, were captured entirely by a camera to ensure that the performed activities are apparent to the annotators. In addition to the 14 subjects, the RGB camera recorded other people who were in the test field at the same time. They provided guidance when the task was unclear, ensured that none of the markers or sensors detached and continuously maintained the experimental setup e.g., by refilling the shelves with packed goods. In addition, photos taken before and after the recordings are included in the protocol.

The Remarks section in the protocol includes the number and time of the breaks taken by the subjects, re-calibration of the OMoCap system during the session, injuries of the subjects and unusual movement during recording, e.g., drinking. Incidents mainly include lost or shifted markers and sensors. If a loss was observed during a recording, it was aborted, deleted, and restarted from the beginning. In three instances, a detachment was noticed after the recording session:

- Incidents with respect to S11: After recording 27, it was noticed that the marker of the left finger (see Figure 8, marker number 22) was misplaced. The reseach group could not determine when exactly the marker shifted its position. After recording 30, it was noticed that the marker of the right ankle (see Figure 8, marker number 35) was lost.
- Incidents with respect to S13: After the last recording (number 30), it was noticed that the marker from the right finger (see Figure 8, marker number 23) and the marker from the left wrist (see Figure 8, marker number 18) were missing. One of the lost markers was found on the left side of the subject’s chest.
- Incidents with respect to S14: After recording number 15, it was noticed that the marker of the right forearm (see Figure 8, marker number 17) was stuck to the leg. For the subsequent recordings (number 16 to 30), the marker was put back to its proper position.

Despite these incidents, the data acquired through these recordings were found to be usable.

### 3.6. Classes and Attributes

This subsection explains the definitions of human activities in the dataset. The dataset considers periodic and static activities, following [1]. The dataset contains annotations of semantic coarse-descriptions of the activities. These semantic definitions are called attributes and they are motivated by HAR methods in [13,16]. An attribute representation can be seen as an intermediate binary-mapping between sequential data and human activities. This intermediate mapping is beneficial for solving HAR problems because they allow sharing high-level concepts among the activity classes. The consequences of unbalanced class-problem can be reduced. A dataset for HAR contains a set of *N* sequential samples $X=x_{1},x_{2},\ldots ,x_{N}\in R^{D}$—for **LARa** dataset either the OMoCap or the IMUs. *D* represents the number of joints or sensors for each dimension [x,y,z]. This parameter is also addressed as the number of sequence channels; their respective activity classes $Y^{c}=y_{1}^{c},y_{2}^{c},\ldots ,y_{N}^{c}\in N$ from a set of *C* activity classes. Following the method in [16], this dataset provides additionally attribute annotations $Y^{a}=y_{1}^{a},y_{2}^{a},\ldots ,y_{N}^{a}$, where $Y^{a}$ is drawn from an attribute representation $A_{\left[K,M\right]}\in B^{K}$. *A* is a binary attribute-representation of size [K,M], with *M* number of attribute representations of size *K* for all of the activity classes. A single attribute representation $y^{a}$ serves as an intermediate representation between an input signal x∈X and the expected activity class $Y^{c}$, i.e., $x\to Y^{a}\to Y^{c}$. There are *M* different attribute representations. This is different from [16], where the authors assign a single, random attribute-representation to an activity class. In this work, the number *M* of representations is stated after the annotation process. In the annotation process, a set of attributes are given to short windows of the recordings, concerning the human movements. Table 7 shows the number of different attribute representations per activity class in **LARa**.

The definition of activities and their semantic attributes is derived from the researchers’ experience [13,33], and from HAR methods [1,16]. The attributes’ and activities’ terminology by default implies industrial context. This excludes irrelevant activities for warehousing, such as smoking or preparing coffee. This is referred to as a Closed-World Condition [34].

#### 3.6.1. Activity Classes

There are eight $C=c_{1},\ldots ,c_{8}\in N^{8}$ activity classes, see Table 2. *Standing, Walking* and *Cart* emphasize the subject’s locomotion. The *Handling* activities refer to a motion of the arms and hands when manipulating an article, box, or tool. These activities do not consider holding an element while standing or walking. *Synchronization* is crucial for proper annotation and for transferring the labels to different sensor streams.

#### 3.6.2. Attributes

**Figure 11:**
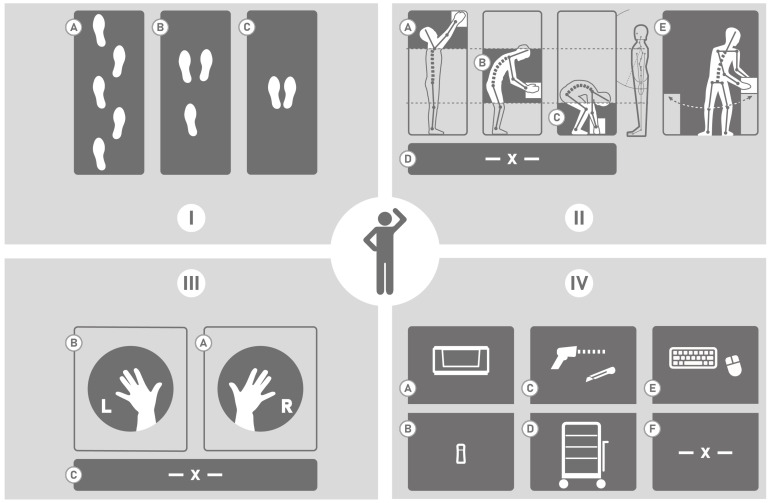
Semantic attributes.

There are K=19 attributes $A\in B^{K}$. These are coarse-semantic descriptions of the activities. They are mostly related to the locomotion and the pose when moving. The human pose changes according to handling different elements and to different heights. The attributes are subdivided in five groups, see Table 3 and Figure 11.

During the labeling, annotators follow these rules: at least one for the attributes per group must be assigned; In group I, the attributes are disjoint, since a subject performs either one of the motions at the same time; The attributes A-D of group II are disjoint while the torso rotation is independent. In the third group, the choice between right and left is non-exclusive as one can use both arms at the same time. In group IV, the attributes are disjoint. Annotators give priority to the items according to a hierarchy: Utility−Auxiliary→Computer→HandyUnit→BulkyUnit→Cart; the *None* and the *Synchronization* classes have a fixed attribute representation. The execution of the waving motion for synchronizing is predefined.

#### 3.6.3. Exemplary Activity Sequence and Its Proper Annotation

Table 4 shows an exemplary warehousing process that consists of four process steps. This process is an excerpt from Scenario 2. In the first process step, the subject is initially standing (Act. 1) before walking to the cart without holding anything in hands (Act. 2). Then, the cart is brought into proper position with both hands while performing smaller steps (Act. 3) and the subject pulls the cart to the retrieval location using the right hand (Act. 4).

By the beginning of the process step 2, the subject is standing while resting the right hand on the cart’s handle (Act. 5). Then the subject proceeds to take the scanner from the cart. The first half of this left-handed handling motion is done while performing a step (Act. 6), while the latter is performed while standing with both feet on the ground (Act. 7). It is important to note that the scanner is annotated as a *Handy Unit* because it is handled as such. In contrast, using it in the following activity is annotated with *Utility Auxiliary*. The label is located on the subject’s right and on eye level so a *Torso Rotation* is necessary and the handling is performed *upwards* (Act. 8). The ninth activity refers to the subject mounting the scanner back on the cart (Act. 9).

In the third process step, the subject picks the item from the shelf (Act. 10–12) and places it in a box located on the lowest level of the cart (Act. 13 and 14). Finally, the pick is confirmed by clicking the put-to-light button located above the box (Act. 15).

There is a wide variety of activity sequences that may constitute the same process. For example, different subjects use different hands when handling an element. In addition, their body motions differ when lifting something from the same height depending on their body size. Thus, the exemplary sequence of activities in Table 4, their class labels and attribute representation are one of many viable options.

### 3.7. Annotation and Revision

**Figure A3:**
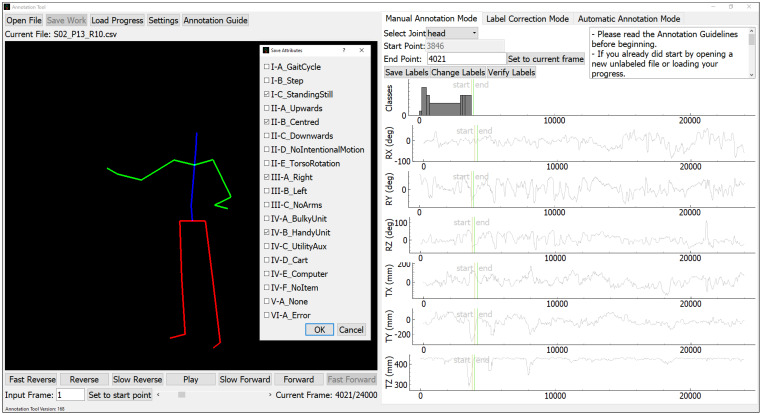
Screenshot of the annotation and revision tool during the annotation.

A Python tool was created for annotating the OMoCap data, see Figure A3. The procedure of the annotation and revision is described by Reining et al. [38]. The annotation tool offers a visualization of the skeleton from the OMocap data and a window-based annotation frame. A window is a segment that is extracted from the sequential data. In the annotation process, an annotator provides the activity class and the attribute representation of a window. Window sizes are variable. The annotator selects consequently the size of a window. Twelve annotators labeled the OMoCap data of the 14 subjects. Apart from two annotators, none of them had any prior experience regarding the annotation of OMoCap data. Each annotator followed the guidelines, as mentioned in Section 3.6. Additionally, RGB videos served as an additional aid for complex activities.

The total time effort for annotation comprised over 474 PHR (19.75 days or 0.65 months). Table 5 illustrates the annotation effort per individual annotator. The information given in the table relates to two-minute recordings. With a range of 39 min to almost 3 h of annotation per recording, the annotators differ greatly in their annotation speed. The reasons for the different annotation speeds are the different level of experience of the annotators, the different setting of window sizes of activities and the individually selectable playback speed of the OMoCap recordings in the annotation tool. An average of 37.5 min was required for a one-minute recording.

Following the annotation, data were revised by four domain experts, see Table 6. The revision of an annotated two-minute recording varied between 4 and 121 min, depending on the quality of the annotation. Compared to the annotation, the average time for a revision is significantly lesser at 11.19 min for a one-minute recording.

The dataset is unbalanced. The *Handling* classes represent nearly 60% of the recordings. These classes show also a higher variability of their attribute representations; this means that these classes show up in many different forms. The class *Handling (centered)* is the most frequent activity by far.

The representations of the *Walking* activity class differ in regards to the handedness and Item pose. This is because the *Gait Cycle* and the *No Intentional Motion* attribute are fixed. The third class *Cart* can only have three representations. Either the cart is pushed or pulled using the *Left Hand*, the *Right Hand*, or with both hands while walking. By definition, there is only one valid representation for both *Synchronization* and *None* classes. This is reflected in the results of the annotation and revision, see Table 7.

### 3.8. Folder Overview of the LARa Dataset

**LARa** contains data of an OMoCap system, one IMU-set, and one RGB camera as well as the recording protocol, the tool for annotation and revision and the networks of activity classes and attributes. Table 8 illustrates an overview of the sizes of the folders and the formats of the files.

The files of the OMoCap data, IMU data, and RGB videos are named after the logistics scenarios, subject, and recording. For example the file name L01_S02_R12 means logistics scenario 01, subject 02, recording 12.

## 4. Deploying LARa for HAR

**Figure 12:**
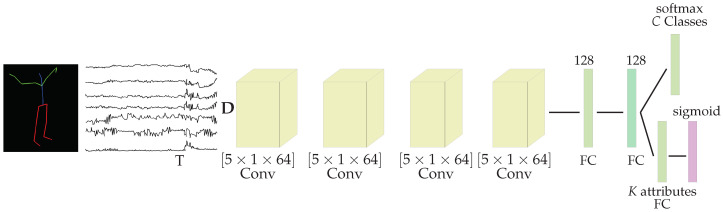
The Temporal Convolutional Neural Network (tCNN) architecture contains four convolutional layers of size [5×1×64]. According to the classification task, there are two types of last fully-connected layer: a softmax and a sigmoid.

The tCNN, proposed in [18], was deployed for solving HAR using the **LARa** dataset. Some minor changes on the architecture are, here, proposed. Our tCNN contains four convolutional layers, no downsampling operations, and three fully-connected layers. Downsampling operations are not deployed as they affect the performance of the network negatively following the conclusions of [16]. The convolutional layers are composed of 64 filters of size [5,1], which perform convolutions along the time axis. The first and second fully connected-layers contain 128 units. Considering the definitions in Section 3.6, there are two different last fully connected layers, depending on the task. A softmax layer is used for direct classification of the activity classes. It has C=8 units. A fully connected layer with sigmoid activation function is used for computing attributes. This layer contains 19 units. The number of output units corresponds to either the number of classes or attributes, respectively, see Section 3.6. Figure 12 shows the tCNN’s architecture.

The architecture processes sequence segments that consist of a feature map input of size [T,D], with *T* the sequence length and *D* the number of sequence channels. The sequence segments are extracted following a sliding-window approach with window size of T=200, step size of s=25 (87.5% overlapping). The number of sequence channels *D* is 126, as there are measurements of position and rotation in [x,y,z] for the 21 joints of the **LARa** OMoCap dataset. This excludes the joint “lower_back” as it is used for normalizing the human poses with respect to the subject. In general, the input sequence is [T=200,D=126] for the dataset. The tCNN computes, either, an activity class $y^{c}$ or a binary-attribute representation $y^{a}$ from an input sequence. Predicting attribute representation follows the method in [16]. Differently from a standard tCNN, this architecture contains a sigmoid activation function replacing the softmax layer. The sigmoid activation function is computed as $sidmoid\left(x\right)=\frac{1}{1+e^{-x}}$. This function is applied to each element of the output layer. The output $\tilde{y^{a}}\in B^{19}$ can be considered as binary pseudo-probabilities for each attribute being present or not in the input sequence.

The architecture is trained using the binary-cross entropy loss given by:

(1) $$E\left(y^{a},\tilde{y^{a}}\right)=-\frac{1}{n}\sum_{i=1}^{n}\left(y_{i}^{a}log\tilde{y_{i}^{a}}+\left(1-y_{i}^{a}\right)log\left(1-\tilde{y_{i}^{a}}\right)\right),$$

with $y^{a}\in B^{19}$ the target attribute representation and $\tilde{y^{a}}\in B^{19}$ the output of the architecture.

Following [3,12], input sequences are normalized per sensor channel to the range of [0,1]. Additionally, a Gaussian noise with parameters [μ=0,σ=0.01] is added. This noise simulates sensor’s inaccuracies.

Following the training procedures from [1,2], the **LARa** OMoCap is divided into three sets: the training, validation, and testing. The training set comprises recordings from subjects [S01,S02,S03,S04,S07,S08,S09,S10]. The validation and testing sets are comprised of recordings from [S05,S11,S12] and [S06,S13,S14], respectively. An early stopping approach is followed using the validation set. This set also is deployed for finding proper training hyperparameters. Recordings with label *None* are not considered for training following the procedure in [3]. The architecture is trained using the batch gradient-descent with RMSProp update rule with an RMS decay of 0.9, a learning rate of $1\times 10^{-5}$, and a batch size of 400. Moreover, Dropout was applied to the first and second fully-connected layers.

In the case of predicting attributes and for solving HAR, a nearest neighbor (NN) approach was used for computing a class c∈C. The Euclidean distance is measured from the predicted attribute vector $\tilde{y^{a}}$ to attribute representation $A\in B^{\left[M,K\right]}$, with M=204 and K=19. This is possible as each activity class c∈C is related to a certain number of binary-attributes vectors in the attribute representation *A*, see Table 7. **LARa** provides the attribute presentation *A*. Both the attribute vector $\tilde{a}$ and the attribute representations *A* are normalized using the 2-norm. The tCNN is also trained using a softmax layer predicting activity classes directly. In this case, the architecture is trained using the Cross-Entropy Loss.

Table 9 and Table 10 present the performance of the method solving HAR on the **LARa** OMoCap dataset using the softmax layer and the attribute representation. Precision is computed as $P=\frac{TP}{TP+FP}$. Recall is computed as $R=\frac{TP}{TP+FN}$. Having TP, FP, FN as the true positives, false positives, and false negatives. The weighted F1 is calculated as $wF1=\sum_{i}^{C}2\times \frac{n_{i}}{N}\times \frac{P_{i}\times R_{i}}{P_{i}+R_{i}}$, with $n_{i}$ being the number of window samples of class $C_{i}\in C$. *Handling* and moving *Cart* activities show the best performances. Using the attribute representation boost the performance in comparison with the softmax classifier. The approach classifies the *Synchronization* and *Standing* activities when using attribute representations. In general, deploying an attribute representations boosts the performance of HAR. These results coincide with [13,16]. Attributes belonging of frequent classes help with the classification of less frequent classes. The effects of the unbalanced problem are also reduced.

Table 11 and Table 12 show the confusion matrices of the predictions using the tCNN in combination with: the softmax layer and the NN using the attribute representation. In general, the method exhibits difficulties predicting the class *Standing*. The method mispredicts *Standing* sequence segments as *Handling (centered)* ones. The class *Walking* present also some mispredictions. Following the results on Table 9 and Table 10, solving HAR using the attribute representation offers a better performance in comparison with the usage of a softmax layer. The classification of activity classes, e.g., *Synchronization*, *Standing*, and *Walking*, improve significantly.

Table 13 presents the performance on the attributes. Attributes are correctly classified in general. Attributes *none* and *error* are not present in the test dataset. However, they are not misclassified. Attribute *Torso Rotation* is also not mispredicted. Nevertheless, the precision and recall of this attribute are zero. This suggests that it is not classified when it shall be. Further, an improvement in this particular attribute is needed.

Both trained tCNNs (using a softmax and a sigmoid layer) and the attribute representation *A* are included in the annotation and revision tool. Implementation code of the annotation tool is also available in [39]. These results seek to give a first evaluation of the dataset for solving HAR.

## 5. Discussion and Conclusions

This contribution presents the first freely accessible dataset for the sensor-based recognition of human activities in logistics using semantic attributes, called **LARa**. Guidelines for creating a dataset were developed based on an analysis of related datasets. Following these guidelines, 758 min of picking and packing activities of 14 subjects were recorded, annotated, and revised. The dataset contains OMoCap data, IMU data, RGB videos, the recording protocol, and the tool for annotation and revision. Multichannel time-series HAR was solved for **LARa** using temporal convolutional neural networks (tCNNs). Classification performance is consequent to the state-of-the-art using tCNNs. Semantic descriptions or attributes of human activities improve classification performance. This supports the effort of annotating attributes and the conclusions from [16].

From an application perspective, the following approaches for fundamental research as well as industrial application result from the **LARa** dataset:

- The laboratory dataset **LARa** will be deployed on IMU data recorded in an industrial environment. The addition of more subjects and the inclusion of further logistical processes and objects is conceivable. New attributes may be added.
- Another approach to recognize human activity is the context. The context may provide information about locations and articles and broaden the application spectrum of the dataset. Context information about the process is provided in this contribution.
- Dependencies between the activities have to be examined, e.g., state-machines. Can information about dependencies increase the accuracy of the recognition of human activities in logistics?
- Finally, the industrial applicability must be proven through a comparison between sensor-based HAR and manual-time management methods, such as REFA and MTM. Can manual-time management methods be enhanced using HAR and **LARa**?

Further experiments concerning the relation among the activity classes and the attributes will be relevant to evaluate. Analyzing the architecture’s filters and their activations using the attribute representations will be useful for understanding how the deep architectures process input signals. **LARa** dataset can be used for solving retrieval problem in HAR. Retrieval tasks might help facilitate data annotation. Additionally, data-stream-approaches will be relevant to be addressed using this dataset. A comparison of HAR methods using statistical pattern recognition and deep architectures is to be also addressed. The extensive **LARa** dataset will be of use for investigating RNNs. Computational causal behavior models are of interest for including the flow charts of the scenarios and longer temporal relations of the input signals.

## Figures and Tables

**Table 1:** Subject: specifications and scenario assignment.

ID	Sex	Age	Weight	Height	Handedness	OMoCap	IMU-set			Scenario 1	Scenario 2	Scenario 3
	[F/M]	[year]	[kg]	[cm]	[L/R]		[1]	[2]	[3]	[Number of Two-Minute Recordings]		
S01	M	28	78	175	L	x	x			29	0	0
S02	F	24	62	163	L	x	x			30	0	0
S03	M	59	71	171	R	x	x			27	0	0
S04	F	53	64	165	L	x	x			29	0	0
S05	M	28	79	185	R	x	x			26	0	0
S06	F	22	52	163	R	x	x			30	0	0
S07	M	23	65	177	R	x		x	x	2	13	14
S08	F	51	68	168	R	x		x	x	2	13	14
S09	M	35	100	172	R	x		x	x	2	14	13
S10	M	49	97	181	R	x		x	x	2	13	12
S11	F	47	66	175	R	x		x	x	2	12	0
S12	F	23	48	163	R	x		x	x	0	6	14
S13	F	25	54	163	R	x		x	x	2	14	14
S14	M	54	90	177	R	x		x	x	2	14	14
**Min.**		**22**	**48**	**163**					
**Avg.**		**37**	**71**	**171**					
**Max.**		**59**	**100**	**185**					
**Sum**										**185**	**99**	**95**

**Table 2:** Activity Classes and their semantic meaning.

Activity Class		Description
$c_{1}$	Standing	The subject is standing still on the ground or performs smaller steps. The subject can hold something in hands or stand hands-free.
$c_{2}$	Walking	The subject performs a gait cycle [35] (pp. 3–7) while carrying something or the subject is walking hands-free. The only exception is made in regards to a cart (see below).
$c_{3}$	Cart	The subject is walking (gait cycle) with the cart to a new position. This class does not include the handling of items on the cart like putting boxes or retrieving items. Likewise, the handling of the cart, e.g., turning it to better reach its handles, is not included.
$c_{4}$	Handling (upwards)	At least one hand reaches the height of the shoulder height (80% of a person’s total height [36] (p. 146)) or is lifted beyond that during the handling activity.
$c_{5}$	Handling (centred)	Handling is possible without bending over, kneeling, or lifting arms to shoulder joint height.
$c_{6}$	Handling (downwards)	The hands are below the height of the knees (lower than 30% of a person’s total height [36] (p. 146)). The subject’s spine is horizontal or they are kneeling.
$c_{7}$	Synchronization	Waving Motion where both hands are above the subject’s head by the beginning of each recording.
$c_{8}$	None	Excerpts that shall not be taken into account, because the class is not recognisable. Reasons are errors or gaps in the recording or a sudden cut by the end of a recording unit.

**Table 3:** Attributes and their semantic meaning.

Attribute		Description
**I - Legs**		
**A**	Gait Cycle	The subject performs a gait cycle [35] (pp. 3–7).
**B**	Step	A single step where the feet leave the ground without a foot swing [35] (pp. 3–7). This can also refer to a step forward, followed by a step backwards using the same foot.
**C**	Standing Still	Both feet stay on the ground.
**II - Upper Body**		
**A**	Upwards	At least one hand reaches the height of the shoulder height (80% of a person’s total height [36] (p. 146)) or is lifted beyond that during the handling activity.
**B**	Centred	Handling is possible without bending over, kneeling or lifting arms to shoulder joint height.
**C**	Downwards	The hands are below the height of the knees (lower than 30% of a person’s total height [36] (p. 146)). The subject’s spine is horizontal or they are kneeling.
**D**	No Intentional Motion	Default value when no intentional motion is performed, e.g., when standing without doing anything, carrying a box or walking with a cart. This is because there is no intentional motion when performing these activities, only a steady stance.
**E**	Torso Rotation	Rotation in the transverse plane [37] (pp. 2–3). Either a rotating motion, e.g., when taking something from the cart and turning towards the shelf or a fixed position when handling something while the torso is rotated.
**III - Handedness**		
**A**	Right Hand	The subject handles or holds something using the right hand.
**B**	Left Hand	The subject handles or holds something using the left hand.
**C**	No Hand	Hands are not used, neither for holding nor for handling something.
**IV - Item Pose**		
**A**	Bulky Unit	Items that the subject cannot put the hands around, e.g., boxes.
**B**	Handy Unit	Items that can be carried with a single hand or that the subjects can put their hands around, e.g., small articles, plastic bags.
**C**	Utility Auxiliary	Use of equipment, e.g., scissors, knives, bubble wrap, stamps, labels, scanners, packaging tape dispenser, adhesives etc.
**D**	Cart	Either bringing the the cart into proper position before taking it to a different location (*Handling*) or walking with the cart to a new location (*No Intentional Motion*).
**E**	Computer	Using mouse and keyboard.
**F**	No Item	Activities that do not include any item, e.g., when the subject fumbles for something when on the search for a specific item.
**V - None**		
**A**	None	Equivalent to the *None* class.

**Table 4:** Exemplary picking process broken down into process steps, activities, classes, and attributes.

				Attribute Representation																
				I Legs			II Upper Body					III Hand.			IV Item Pose					
				Gait Cycle	Step	Standing Still	Upwards	Centered	Downwards	No Intentional Motion	Torso Rotation	Right Hand	Left Hand	No Hand	Bulky Unit	Handy Unit	Utility/Auxiliary	Cart	Computer	No Item
Process Step		Act.	Class	A	B	C	A	B	C	D	E	A	B	C	A	B	C	D	E	F
**1**	Bring cart to	1	$c_{1}$ Standing	0	0	1	0	0	0	1	0	0	0	1	0	0	0	0	0	1
	retrieval	2	$c_{2}$ Walking	1	0	0	0	0	0	1	0	0	0	1	0	0	0	0	0	1
	location	3	$c_{5}$ Hand. (cen.)	0	1	0	0	1	0	0	0	1	1	0	0	0	0	1	0	0
		4	$c_{3}$ Cart	1	0	0	0	0	0	1	0	1	0	0	0	0	0	1	0	0
**2**	Scan Barcode	5	$c_{1}$ Standing	0	0	1	0	0	0	1	0	1	0	0	0	0	0	1	0	0
		6	$c_{5}$ Hand. (cen.)	0	1	0	0	1	0	0	0	0	1	0	0	1	0	0	0	0
		7	$c_{5}$ Hand. (cen.)	0	0	1	0	1	0	0	0	0	1	0	0	1	0	0	0	0
		8	$c_{4}$ Hand. (upw.)	0	0	1	1	0	0	0	1	0	1	0	0	0	1	0	0	0
		9	$c_{5}$ Hand. (cen.)	0	1	0	0	1	0	0	0	0	1	0	0	1	0	0	0	0
**3**	Retrieve item	10	$c_{4}$ Hand. (upw.)	0	1	0	1	0	0	0	0	1	0	0	0	1	0	0	0	0
	and put in	11	$c_{4}$ Hand. (upw.)	0	0	1	1	0	0	0	0	1	0	0	0	1	0	0	0	0
	box	12	$c_{4}$ Hand. (upw.)	0	1	0	1	0	0	0	0	1	0	0	0	1	0	0	0	0
		13	$c_{6}$ Hand. (down.)	0	1	0	0	0	1	0	0	1	1	0	0	1	0	0	0	0
		14	$c_{6}$ Hand. (down.)	0	0	1	0	0	1	0	0	1	1	0	0	1	0	0	0	0
**4**	Confirm pick	15	$c_{6}$ Hand. (down)	0	0	1	0	0	1	0	0	1	0	0	0	0	1	0	0	0

**Table 5:** Annotation effort of all annotators.

ID	Total Time	No of Rec.	Time per Rec.
	[hh:mm:ss]		[hh:mm:ss]
A01	55:12:19	52	01:14:02
A02	73:22:04	45	01:55:21
A03	56:30:39	54	01:14:13
A04	34:39:08	26	01:28:00
A05	84:18:37	30	02:48:37
A06	39:24:16	64	00:39:46
A07	28:40:57	25	01:10:35
A08	32:56:40	27	01:15:24
A09	33:28:45	27	01:14:24
A10	10:14:21	12	00:51:12
A11	23:03:16	14	01:38:48
A12	02:16:00	3	01:45:03
**Min.**			**00:39:46**
**Max.**			**02:48:37**
**Sum**	**474:07:02**	**379**	

**Table 6:** Revision effort of all revisers.

ID	Total Time	No of Rec.	Time per Rec.
	[hh:mm:ss]		[hh:mm:ss]
Re01	13:44:00	88	00:09:22
Re02	39:18:00	97	00:24:19
Re03	28:37:00	91	00:18:52
Re04	61:19:00	103	00:35:43
**Min.**			**00:09:22**
**Max.**			**00:35:43**
**Sum**	**142:58:00**	**379**	

**Table 7:** Annotation results divided by activity classes.

	Stand.	Walk.	Cart	Handling (upwards)	Handling (centred)	Handling (downwards)	Synchron.	None
**Samples**	974,611	994,880	1,185,788	754,807	3,901,899	673,655	158,655	403,737
**Avg. Time/Occ. [s]**	1.71	3.72	6.46	2.72	4.39	2.74	2.16	7.10
**Proportion [%]**	10.77	11.00	13.11	8.34	43.12	7.45	1.75	4.46
**[M] number of Attr. representations**	28	7	3	45	72	47	1	1

**Table 8:** Folder overview of the LARa  dataset.

Folder	Folder Size [MiB]	File Format	Recording Rate
OMoCap data	33,774	csv	200 fps
IMU data - MbientLab	1355.77	csv	100 Hz
RGB videos	17,974.82	mp4	30 fps
recording protocol	2.58	pdf	-
annotation and revision tool	2899.99	py	-
class_network	1449.55	pt	-
attrib_network	1449.55	pt	-

**Table 9:** Recall [%] and precision [%] of human activity recognition (HAR) on the LARa OMoCap dataset.

Output	Metric	Performance						
		Stand.	Walk.	Cart	Hand. (up.)	Hand. (cent.)	Hand. (down.)	Sync.
Softmax	Recall[%]	3.11	71.96	71.34	61.39	87.40	65.30	0.0
	Precision[%]	73.00	45.29	81.35	57.10	70.85	80.72	0.0
Attributes	Recall[%]	55.86.	54.31.	76.12	69.16	80.99	74.36	69.84
	Precision[%]	24.22	60.59	92.13	79.08	82.94	74.63	89.31

**Table 10:** The over-all accuracy [%] and weighted F1 [%] of HAR on the LARa OMoCap dataset.

Metric	Perform.	
	Softmax	Attributes
Acc[%]	68.88	75.15
wF1[%]	64.43	73.62

**Table 11:** Confusion matrix from the class predictions using tCNN with the softmax layer.

Activities	Confusion Matrix						
	Stand.	Walk.	Cart	Hand. (up.)	Hand. (cent.)	Hand. (down.)	Sync.
Stand.	**311**	1807	211	134	7446	86	0
Walk.	42	**3776**	461	35	918	15	0
Cart	1	928	**9005**	4	2684	1	0
Hand. (up.)	0	92	152	**4188**	2389	1	0
Hand. (cent.)	72	1681	1233	1717	**36,587**	572	0
Hand. (down.)	0	51	2	12	1437	**2826**	0
Sync.	0	2	6	1245	178	0	**0**

**Table 12:** Confusion matrix from the class predictions using the attribute predictions with tCNN and the nearest neighbor (NN) approach.

Activities	Confusion Matrix						
	Stand.	Walk.	Cart	Hand. (up.)	Hand. (cent.)	Hand. (down.)	Sync.
Stand.	**2421**	1492	506	268	4668	219	421
Walk.	633	**3179**	691	47	649	41	7
Cart	44	298	**11,630**	20	629	2	0
Hand. (up.)	71	44	32	**5395**	1218	20	42
Hand. (cent.)	1085	825	2403	1919	**34,719**	832	79
Hand. (down.)	73	15	16	9	982	**3230**	3
Sync.	7	0	0	143	3	0	**1278**

**Table 13:** The accuracy, precision, and recall [%] for the attributes on the test dataset.

Metric	Attributes																		
	$a^{1}$	$a^{2}$	$a^{3}$	$a^{4}$	$a^{5}$	$a^{6}$	$a^{7}$	$a^{8}$	$a^{9}$	$a^{10}$	$a^{11}$	$a^{12}$	$a^{13}$	$a^{14}$	$a^{15}$	$a^{16}$	$a^{17}$	$a^{18}$	$a^{19}$
Accuracy	89.3	76.9	84.5	93.9	81.7	96.4	82.5	96.9	92.0	79.1	90.3	76.2	71.7	85.2	91.3	98.3	90.2	100	100
Precision	79.0	82.8	83.4	80.4	85.6	76.7	86.3	0.0	92.8	81.6	91.9	48.7	60.4	74.3	88.8	98.8	95.4	0.0	0.0
Recall	82.1	70.3	92.0	73.1	83.9	68.7	72.0	0.0	98.5	92.4	36.2	37.2	63.0	26.8	74.2	49.5	41.0	0.0	0.0

**Figure A1:**
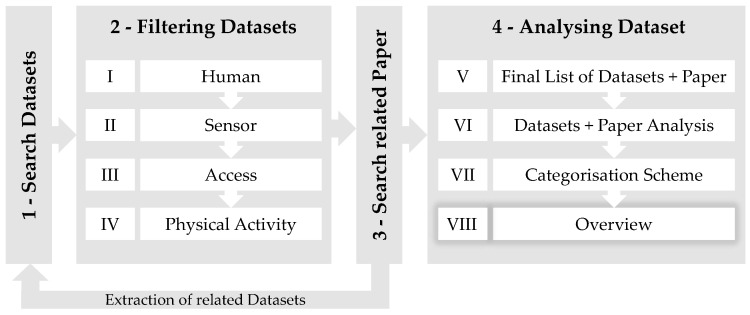
Method of the dataset overview.

**Figure A2:**
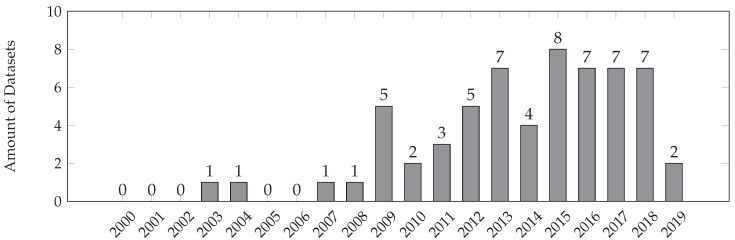
Datasets by year of publication.

## Appendix A. Related Datasets

This section gives an overview of state-of-the-art datasets for HAR. Moreover, it underlines the necessity of a new dataset for HAR in logistics. Similarities from different datasets help as a base for creating a new guidelines. The guidelines describe how a dataset for HAR is created, answering the second research question. The novel **LARa** dataset is created based on the new guidelines and the findings gained from a similar dataset to logistics. Due to data protection regulations, the following analysis is restricted to OMoCap and IMU datasets.

### State-of-the-Art Datasets for HAR

A dataset overview is followed. This overview is a modification of the guidelines for Literature Reviews suggested by Reining et al. [10], Kitchenham and Brereton [40,41,42], and Chen et al. [43]. Figure A1 illustrates the dataset overview steps. Firstly, datasets are searched according to a predefined list of keywords. Secondly, the list of datasets is filtered by Content Criteria. Thirdly, datasets are obtained from papers that present methods for HAR. This step includes a loop. Fourthly, the content and properties of the datasets are analyzed.

The search process with predefined keywords was carried out on the following platforms: Figshare, Github, Google Scholar, Researchgate, Science Direct, Scopus, UCI Machine Learning Repository, and Zenodo. Only datasets presented in English were taken into consideration during this process. All datasets must have been published by 31st March 2020. While collecting the dataset, the keywords used for the search consisted of three categories:

- Keyword Category A: dataset, data set, database, challenge, library, repository
- Keyword Category B: IMU, inertial measurement unit, accelerometer, MoCap, OMoCap, motion capturing, sensor
- Keyword Category C: HAR, human activity recognition, human activity/activities/actions/ movements/gestures

The platforms were searched with the keywords from Category A in combination with the keywords from Category B or C: e.g., IMU dataset, HAR dataset, or accelerometer database.

A filtering process reduces the list of searched datasets. This process is based on a predefined list of keywords, Table A1, and it is divided into four stages, see Figure A1. At each stage, a dataset will be examined if it includes one of the content criteria consecutively. If the dataset does not meet any of the Content Criteria, it will be excluded from the following steps. Datasets in Stage IV were taken into account for further analysis.

**Table A1:** Content criteria for filtering process.

Stage	Content Criteria	Description
**I**	Human	Data must relate to human movements.
**II**	Sensor	Dataset must contain IMU or OMoCap data, or both.
**III**	Access	Dataset must be accessible online, downloadable and free of charge.
**IV**	Physical Activity	Caspersen et al. [44] defined physical activity “as any bodily movement produced by skeletal muscles that results in energy expenditure”. The definition of physical activity is limited by torso and limb movement [10].

Stage I contains 173 unique datasets that consider the recognition of human movements. In Stage II, 78 datasets were excluded as these do not consist of measurements from IMU or OMoCap. They are based mainly on RGB and depth data. Moreover, datasets with inertial measurements from objects are also excluded. 25 datasets are excluded in Stage III as their accesses are restricted or not possible. Unreachable URLs are the common cause. Moreover, datasets, which required a paid account or a one-time payment or with downloading errors, are not considered. In Stage IV, 9 datasets are excluded as they do not include human activities according to Caspersen’s definition [44] and to the limitation of the bodily movement to torso and limb movement. These activities relate to emotion, gender identification, occupancy detection, or facial expression. Furthermore, simulations are excluded. At this Stage, unlabeled datasets are also excluded.

A total of 61 datasets corresponded to all four Content Criteria. Table A2 shows the filtering stages.

**Table A2:** Examined datasets per stage.

Stage	Content Criteria	No of Datasets
**I**	Human	173
**II**	Sensor	95
**III**	Access	70
**IV**	Physical Activity	61

Scientific publications from the datasets that describe the datasets in detail are searched. They also offer applications of the datasets solving different problems. These publications became additionally a source of identifying related datasets, as they tend to compare them.

Datasets and the corresponding publications from Stage IV are organized according to a categorization scheme. It consists of five categories: General information, Domain, Data Specification, Sensor type, and sensor location. Table A3 presents four of these categories. Table A4 organizes the datasets following the categorization scheme. The fifth root category refers to the attachment of the sensors and markers to the subject’s body, divided into 13 body regions. Key information regarding the annotation were noted. This includes the annotation method, number and area of the annotators’ expertise, the annotation effort, the annotation tool, and supporting sensors.

Analyzing the Figure A2, there has been a significant increase in published datasets since 2009. There were no time frame restrictions considered during the filtering process. However, no records could be found before 2003 and only four were found in the period, 2003 to 2008.

**Table A3:** Categorization scheme.

Root Category		
	Subcategory	Description
**General Information**		
	Year	Year of publication. Updates are not taken into account.
	Dataset name	Name of the dataset and the acronym
	Ref. [Dataset]	Link and, if available, the Identifier (DOI) of the dataset
	Ref. [Paper]	Identifier or, if not available, link of the paper that describes the dataset, uses it or is generally given as a reference
**Domain of the Act. class**		
	Work	Office work, general work, and physical work in production and logistic
	Exercises	Sport activity classes, e.g., basketball, yoga, boxing, golf, ice hockey, soccer
	Locomotion	e.g., walking, running, elevating, sitting down, going upstairs, and downstairs
	ADL	Activity classes of daily living, e.g., watching TV, shopping, cooking, eating, cleaning, dressing, driving car, personal grooming, interacting, talking, lying
	Fall Detection	Falling in different directions and from different heights
	Hand Gestures	Focus on the movement of hands, e.g., arm swiping, hand waving, and clapping
	Dance	e.g., jazz dance, hip-hop dance, Salsa, Tango
**Data Specification**		
	Recording Time [min]	Total time of the recordings in minutes
	Data Size [MiB]	Data Size of the entire unzipped dataset in mebibytes, including e.g., RGB videos, pictures
	Format	Formats of data published in the repository
	No Subjects	Number of unique subjects
	No Act. classes	Number of individual activity classes
	List Act. classes	List of all individual activity classes
	Laboratory	The recordings were made in a laboratory environment
	Real Life	The recordings were made in a real environment, e.g., outdoors, on a sports field, or in a production facility
**Sensor**		
	OMoCap [Hz]	Optical marker-based Motion Capture with frames per second or hertz as a unit
	IMU [Hz]	Inertial measurement unit with hertz as a unit
	Other Sensors	Sensors except IMU and OMoCap
	Phone, Watch, Glasses	Use of sensors built in smartphone, smartwatch, or smart glasses

Authors give a different meaning to the term activity class. The word activity could describe a movement, an action, a motion, a gesture, and a process step. In some instances, dance movements and gestures have been considered as activities. The overview table shows examples of these instances, such as: Leuven Action Database [45], Vicon Physical Action Dataset [46], Sensors Activity dataset [47], KIT Whole-Body Human Motion Database [48].

Only a few authors have considered a hierarchy of activities and their descriptive features, which are called postures or attributes. The specification in the UTD Multimodal Human Action Dataset [49] is an example. The movements with the left and right hands are regarded as individual activities; thus, the same motion with the opposite hand represents a different activity. Additionally, the duration of the activity differs. Activities may last from a fraction of a second up to several min. For example, the IMU Dataset for Motion and Device Mode Classification in [50] contains activities lasting for 45 to 60 min. The duration of activities in the Physical Activity Monitoring Dataset (PAMAP2) [5] is approximately 3 min.

The recording times vary widely. Without the two longest recordings [51,52], the average recording time per dataset is 603 min. Forty datasets contain 15 or fewer subjects. The number of activity classes that are present in a dataset varies heavily depending on the number and properties of its application domains. For example, [53] solely addressed locomotion activities that were subdivided in *Slowly Walking* and *Running*. In contrast, the dataset presented by Müller et al. contains 70 activity classes in the domains Exercises, Locomotion, ADL, and Dance [54].

Using IMU sensors dominates with 51 datasets. In addition to the significantly lower cost compared to an OMoCap system, its flexible use in a real environment is particularly advantageous. Recording rates vary from 10 Hz [55,56,57] to 700 Hz [58]. With an average rate of 86.2 Hz. The selection of the recording rate is rather arbitrary and not well justified. It is striking that smartphones were used more frequently (24 times) as IMUs. Apart from the smartphones, there are seven datasets with smartwatches [52,59,60,61,62,63,64] and two datasets with smart glasses [62,63]. Further, relying on just IMU data without video recordings would be difficult to annotate, e.g., in non-orchestrated scenarios,. To facilitate the annotation, subjects performed only one activity in one recording, e.g., [65,66]. The realistic body movement as in a daily activity would not be captured in this particular case. Moreover, IMUs tend to be affected by noise in the presence of metal stands and long lasting-recordings due to drift noise. In [67], recordings of 2–6 days per subject were taken; this assumes realistic human activities. From the subject point of view, this could cause fatigue, irritation, and in some cases the actor forgetting to carry/attach the measurement device. The positions of sensors vary. Subjects carry smartphones in their pockets or in their hands. Smartphones have been also placed on the belt. Moreover, one dataset contains recordings from six smartphones. They are distributed over the entire body [51]. The placements may differ from the placements specified in the Table A4. One reason is the difference in the interpretation of body parts. In individual cases, the sensor was placed on the hip and not on the waist or vice versa. If the smartphone was placed in the pocket, the position is regarded as hip or upper leg depending on the position of the pocket.

The OMoCap system requires a complex and cost-intensive infrastructure. A total of 15 datasets have OMoCap data with eleven different recording rates between 30 and 500 Hz. Four datasets contain both IMU and OMoCap data. The attachment of the markers is determined by the respective systems, e.g., provided by Vicon [68]. In addition to IMUs and the OMoCap systems, other sensors were used. In general, RGB video streams are commonly recorded for annotation purposes [4,69,70]. Other sensors are BodMedia, depth-cameras, electromyography (EMG), Global Positioning System (GPS), heart rate monitor, light, infrared, microphone, photoplethysmogram (PPG), pressure sensor hand-glove, and radio-frequency identification (RFID). The OMoCap serves as ground truth in [71,72].

The descriptions of the datasets rarely contain information about annotation. No standardized annotation process can be identified from the available information. There is no common annotation tool and rarely specified, like in [70], which uses the Anvil software [73]. The annotation tool has been published in exceptions such as [48]. The annotations were carried out by both non-domain-experts and domain-experts. In Daphnet Gait, the gait symptom of Parkinson’s patients was analyzed by physiotherapist [69]. In addition to expertise, the number of annotators differs. Hand Gesture dataset [74] was annotated by one person, whereas AndyData-lab-onePerson [70] included three annotators. The specified annotation effort varies greatly. Some data were annotated in real-time [5,69,75,76]. Data such as [4] required 14 to 20 min for the annotation of a one-minute recording.

There is no standardized structure for datasets. Data recorded protocol is not globally predetermined. Likewise, there is no standardized vocabulary. The same term, like *activity class* mentioned before, is understood differently depending on the author. In addition to repositories, datasets are often stored on private websites. As a result of the non-permanent storage, 16 datasets are no longer available. The repositories include: Figshare [77], UCI Machine Learning Repository [78], and Zenodo [79]. Software development and collaborative platforms like GitHub [80] and Dropbox [81], were used for file sharing. Further, ResearchGate [82] is used to save and access datasets as well as to access the papers relevant for the dataset.

Five datasets deal with working activities. The activity classes can be divided into two categories, on the one hand office work or general work such as *“writing on paper”* [64], *“typing on keyboard”* [64], *“working”* [58], *“LAB_WORK”* [52], and on the other hand physical work in production [83] and logistics. *AndyData-lab-onePerson* partially meets logistical activity classes [70]. Maurice et al. [70] had considered ergonomics in an industrial environment with six activities such as screwing in different heights and carrying weights. The annotated movements were divided into three levels. Level one, general posture, includes locomotion. The detailed posture, level two, describes the position of the torso and the hands. Level three, current action, includes movements from intra-logistics and production, such as reach, pick, place, release, carry, manipulation objects, and screw movements in the packaging process, among other things, are missing to fully cover intra-logistics activities.

No dataset from Table A4 meets all the requirements for describing logistical activities, but *AndyData-lab-onePerson* can serve as a blueprint for creating a logistics-dataset.

**Table A4:** Overview of related public available human activity recognition datasets. The entries are sorted chronologically in ascending order according to the year of publication and alphabetically according to the name of the dataset. Missing informations are marked with “-”.

General Information				Domain of the Act. class							Data Specification							Sensor				Attachment (Sensor/Marker)												
Year	Dataset Name	Ref. [Dataset]	Ref. [Paper]	Work	Exercises	Locomotion	ADL	Fall Detection	Hand Gestures	Dance	Recording Time [min]	Data Size [MiB]	Format	No Subjects	No Act. Classes	Laboratory	Real Life	OMoCap [fps/Hz]	IMU [Hz]	Other Sensors	Phone, Watch, Glasses	Hand/Wrist	Lower Arm	Upper Arm	Foot/Ankle	Lower Leg	Upper Leg	Hip	Shoulder	Belly/Waist	Thorax/Chest	Lower Back	Upper Back	Head
2003	Carnegie Mellon University Motion Capture Database (CMU Mocap)	[84]	-		x	x	x			x	-	18,673	amc	112	23	x		120				x	x	x	x	x	x	x	x	x	x	x	x	x
2004	Leuven Action Database	[45]	[85]		x	x	x				-	14	text, avi, xls, pdf	1	22	x		30		RGB		x	x	x	x		x	x	x		x		x	x
2007	HDM05	[54]	[86]		x	x	x			x	-	3000.32	c3d, amc, avi	5	70	x		120		RGB		x	x	x	x	x	x	x	x		x	x	x	x
2008	Wearable Action Recognition Database (WARD)	[87]	[88]			x	x				-	41.66	mat	20	13			x		20			x	x		x	x	x						
2009	BodyAttack Fitness	[83]	[89]		x						15	5.64	mat	1	6	x			64							x	x							
2009	Carnegie Mellon University Multimodal Activity (CMU-MMAC) Database	[59]	[90]				x				-	60,897.03	amc, txt, asf, wav, xls, avi	43	29	x		120	125	RGB, microphone, RFID, BodyMedia	x	x	x	x	x	x	x	x	x		x	x	x	x
2009	HCI gestures	[83]	[89]						x		-	12.9	mat	1	5	x			96				x	x										
2009	HumanEva I	[91]	[92]		x	x					-	13,824	-	4	6	x			120	RGB, depth		x	x	x	x	x	x	x	x		x		x	x
2009	HumanEva II	[93]	[92]			x					-	4,649	-	2	4	x			120	RGB		x	x	x	x	x	x	x	x		x		x	x
2010	KIT Whole-Body Human Motion Database	[48]	[65]		x	x	x		x	x	-	2,097,152	xml, c3d, avi	224	43	x		100		RGB		x	x	x	x	x	x	x	x		x	x	x	x
2010	Localization Data for Person Activity Data Set	[55]	[94]			x	x	x			-	20.5	txt	5	11	x			10						x			x			x			
2011	3DLife/Huawei ACM MM Grand Challenge 2011	[95]	[96]							x	-	-	svl, cvs	15	5	x			160	RGB, microphone, depth			x			x				x				
2011	UCF-iPhone Data Set	[97]	[98]		x	x					-	13.1	csv	9	9		x		60		x							x						
2011	Vicon Physical Action Data Set	[46]	[99]			x	x				33.33	144	txt	10	20	x		200				x	x		x	x								x
2012	Activity Prediction (WISDM)	[100]	[101]			x					-	49.1	txt	29	6	x			20		x						x							
2012	Human Activity Recognition Using Smartphones Data Set (UCI HAR)	[102]	[103]			x	x				192	269	txt	30	6	x			50		x							x						
2012	OPPORTUNITY Activity Recognition Data Set	[4]	[104]			x	x		x		1500	859	txt	12	24		x		32			x	x	x	x	x	x	x				x	x	
2012	PAMAP2 Physical Activity Monitoring Data Set	[5]	[105]		x	x	x				600	1652.47	txt	9	18	x	x		100	heart rate monitor		x			x						x			
2012	USC-SIPI Human Activity Dataset	[76]	[106]			x					-	42.7	mat	14	12		x		100									x						
2013	Actitracker (WISDM)	[107]	[108]			x	x				-	2588.92	txt	29	6		x		20		x						x							
2013	Daily and Sports Activities Data Set	[109]	[110]		x	x	x				760	402	csv	8	19		x		25				x				x				x			
2013	Daphnet Freezing of Gait Data Set	[69]	[111]			x					500	86.2	txt	10	3	x			64	RGB						x	x					x		
2013	Hand Gesture	[74]	[1]				x		x		70	47.6	mat	2	11		x		32			x	x	x										
2013	Physical Activity Recognition Dataset Using Smartphone Sensors	[47]	[112]			x					-	63.1	xlsx	4	6		x		50		x			x			x	x		x				
2013	Teruel-Fall (tFall)	[113]	[114]					x			-	65.5	dat	10	8		x		50		x						x							
2013	Wearable Computing: Accelerometers’ Data Classification of Body Postures and Movements (PUC-Rio)	[56]	[115]			x					480	13.6	dat	4	5	x			10					x		x	x			x				
2014	Activity Recognition from Single Chest-Mounted Accelerometer Data Set	[116]	[117]			x	x				431	44.2	csv	15	7		x		52												x			
2014	Realistic sensor displacement benchmark dataset (REALDISP)	[118]	[119]		x	x					566.02	6717.43	txt	17	33	x			50				x	x		x	x						x	
2014	Sensors activity dataset	[47]	[120]			x					2800	308	csv	10	8		x		50		x		x	x			x	x						
2014	User Identification From Walking Activity Data Set	[121]	[117]			x	x				431	4.18	csv	22	5		x		52	RGB, microphone	x										x			
2015	Complex Human Activities Dataset	[47]	[122]			x	x				390	240	csv	10	13		x		50		x	x					x							
2015	Heterogeneity Activity Recognition Data Set (HHAR)	[60]	[123]			x					270	3333.73	csv	9	6		x		200		x		x					x						
2015	Human Activity Recognition with Inertial Sensors	[57]	[124]			x	x	x			496	324	mat	19	13	x			10			x					x	x						
2015	HuMoD Database	[125]	[126]		x	x					49.4	6044.27	mat	2	8	x		500		EMG					x	x	x	x	x		x	x	x	x
2015	Project Gravity	[61]	[127]			x	x	x			-	27.6	json	3	19	x			25	RGB	x	x					x							
2015	Skoda Mini Checkpoint	[83]	[128]	x					x		180	80.3	mat	1	10		x		98			x	x	x										
2015	Smartphone-Based Recognition of Human Activities and Postural Transitions Data Set (SBHAR)	[129]	[130]			x	x				300	240	txt	30	12		x		50	RGB	x									x				
2015	UTD Multimodal Human Action Dataset (UTD-MHAD)	[49]	[131]		x	x			x		-	1316.15	mat, avi	8	27	x			50	depth			x				x							
2016	Activity Recognition system based on Multisensor data fusion (AReM) Data Set	[132]	[133]			x	x				176	1.69	csv	1	6	x			70		x					x		x			x			
2016	Daily Log	[51]	[134]		x	x	x				106,560	4815.97	csv	7	33		x		x	GPS	x		x				x							
2016	ExtraSensory Dataset	[52]	[135]	x	x	x	x				308,320	144,423.88	dat, csv, mfcc	60	51		x		40	microphone	x	x						x						
2016	HDM12 Dance	[136]	[136]							x	97	2,175.48	asf, c3d	22	20	x		128				x	x	x	x	x	x	x	x		x		x	x
2016	RealWorld	[75]	[67]			x	x				1065	3891.92	csv	15	8		x		50	GPS, magnetic field, microphone, RGB, light	x		x	x		x	x			x	x			x
2016	Smartphone Dataset for Human Activity Recognition in Ambient Assisted Living	[137]	[103]			x	x				94.79	46.5	txt	30	6		x		50		x									x				
2016	UMAFall: Fall Detection Dataset	[138]	[139]			x	x	x			-	359	csv	19	14		x		200		x	x				x		x		x	x			
2017	An Open Dataset for Human Activity Analysis using Smart Devices	[62]	[140]		x	x	x				-	433	csv	1	16		x		x		x	x						x						x
2017	IMU Dataset for Motion and Device Mode Classification	[141]	[50]			x					-	2835.21	mat	8	3		x		100		x	x	x	x	x	x	x	x	x				x	x
2017	Martial Arts, Dancing and Sports (MADS) Dataset	[71]	[142]		x					x	-	24,234.96	mov, zip	5	5	x		60		RGB		x	x	x	x	x	x	x	x		x	x	x	x
2017	Physical Rehabilitation Movements Data Set (UI-PRMD)	[66]	[143]		x	x					-	4700.17	txt	10	10	x		100		depth		x	x	x	x	x	x	x	x		x		x	x
2017	SisFall	[144]	[145]			x	x	x			1849.33	1627.67	txt	38	34		x		200											x				
2017	TotalCapture Dataset	[72]	[146]		x	x					-	-	-	5	5	x		x	60	RGB		x	x	x	x	x	x	x	x		x	x	x	x
2017	UniMiB SHAR	[147]	[148]			x	x	x			-	255	mat	30	17	x			50	microphone	x						x							
2018	Fall-UP Dataset (Human Activity Recognition)	[149]	[150]			x	x	x			165.00	78	csv	17	11	x			100	infrared, RGB		x				x		x	x	x			x	x
2018	First-Person View	[63]	-			x	x				-	1046.86	mp4, csv	2	7		x		x	RGB	x		x								x			x
2018	HAD-AW	[64]	[151]	x	x	x	x			x	-	325	xlsx	16	31		x		50		x	x												
2018	HuGaDB	[152]	[153]			x					600	401	txt	18	12		x		x	EMG					x	x	x							
2018	Oxford Inertial Odometry Dataset (OxIOD)	[53]	[154]			x					883.2	2751.73	csv	4	2	x	x	250	100		x	x						x						
2018	Simulated Falls and Daily Living Activities Data Set	[155]	[156]			x	x	x			630	3972.06	txt	17	36	x			25				x			x	x				x	x		x
2018	UMONS-TAICHI	[157]	[158]		x						-	28,242.47	txt, c3d, tsv	12	13	x		179		RGB, depth		x	x	x	x	x	x	x	x		x		x	x
2019	AndyData-lab-onePerson	[70]	[32]	x		x	x				300	99,803.46	mvn, mvnx, c3d, bvh, csv, qtm, mp4	13	6	x		120	240	RGB, pressure sensor handglove		x	x	x	x	x	x	x	x		x	x	x	x
2019	PPG-DaLiA	[58]	[159]	x		x	x				2,190	23,016.74	pkl, csv	15	8		x		700	PPG, ECG		x									x			
**Sum**	**61**			**5**	**20**	**51**	**35**	**9**	**6**	**7**						**33**	**30**				**25**	**29**	**30**	**24**	**20**	**28**	**36**	**31**	**16**	**10**	**25**	**11**	**18**	**21**
**Min.**											**15**	**1.69**		**1**	**2**			**30**	**10**															
**Avg.**											**13,531.08**	**43,605.15**		**21.1**	**14.8**			**155.9**	**86.2**															
**Max.**											**308,320**	**2,097,152**		**224**	**70**			**500**	**700**															
*2019*	***L**ogistic****A**ctivity****R**ecognition**Ch**a**llenge* (***LARa***)	[160]		*x*		*x*					*758*	*58,907.15*	*csv, mp4, pdf, pt, py*	*14*	*8*	*x*		*200*	*100*	*RGB*		*x*	*x*	*x*	*x*	*x*	*x*	*x*	*x*	*x*	*x*	*x*	*x*	*x*

==

## References

[1] Bulling A., Blanke U., Schiele B. (2014). A Tutorial on Human Activity Recognition Using Body-Worn Inertial Sensors. ACM Comput. Surv. (CSUR).

[2] Ordóñez F.J., Roggen D. (2016). Deep convolutional and LSTM recurrent neural networks for multimodal wearable activity recognition. Sensors.

[3] Grzeszick R., Lenk J.M., Rueda F.M., Fink G.A., Feldhorst S., ten Hompel M. Deep Neural Network based Human Activity Recognition for the Order Picking Process. Proceedings of the 4th International Workshop on Sensor-Based Activity Recognition and Interaction.

[4] Roggen D., Calatroni A., Nguyen-Dinh L.V., Chavarriaga R., Sagha H., Digumarti S.T. Activity Recognition Challenge|Opportunity. http://www.opportunity-project.eu/challenge.html.

[5] Reiss A. (2016). UCI Machine Learning Repository: PAMAP2 Physical Activity Monitoring Data Set. http://archive.ics.uci.edu/ml/datasets/PAMAP2+Physical+Activity+Monitoring.

[6] 2016 Warehouse/DC Operations Survey: Ready to Confront Complexity. https://www.logisticsmgmt.com/article/2016_warehouse_dc_operations_survey_ready_to_confront_complexity.

[7] Schlögl D., Zsifkovits H. (2016). Manuelle Kommissioniersysteme und die Rolle des Menschen. BHM Berg-und Hüttenmännische Monatshefte.

[8] REFA-Time Study. https://refa.de/en/Int.-global-consulting/time-studies.

[9] MTM—Methods-Time Measurement: MTM. https://mtm.org/en/about-mtm/mtm.

[10] Reining C., Niemann F., Moya Rueda F., Fink G.A., ten Hompel M. (2019). Human Activity Recognition for Production and Logistics—A Systematic Literature Review. Information.

[11] Feldhorst S., Masoudenijad M., ten Hompel M., Fink G.A. (2016). Motion Classification for Analyzing the Order Picking Process Using Mobile Sensors—General Concepts, Case Studies and Empirical Evaluation.

[12] Moya Rueda F., Grzeszick R., Fink G., Feldhorst S., ten Hompel M. (2018). Convolutional Neural Networks for Human Activity Recognition Using Body-Worn Sensors. Informatics.

[13] Reining C., Schlangen M., Hissmann L., ten Hompel M., Moya F., Fink G.A. Attribute Representation for Human Activity Recognition of Manual Order Picking Activities. Proceedings of the 5th International Workshop on Sensor-Based Activity Recognition and Interaction—iWOAR ’18.

[14] General Data Protection Regulation (GDPR). https://gdpr.eu/tag/gdpr/.

[15] Venkatapathy A.K.R., Bayhan H., Zeidler F., ten Hompel M. Human Machine Synergies in Intra-Logistics: Creating a Hybrid Network for Research and Technologies. Proceedings of the 2017 Federated Conference on Computer Science and Information Systems (FedCSIS).

[16] Moya Rueda F., Fink G.A. Learning attribute representation for human activity recognition. Proceedings of the 2018 24th International Conference on Pattern Recognition (ICPR).

[17] Ronao C.A., Cho S.B. (2015). Deep convolutional neural networks for human activity recognition with smartphone sensors. Conference on Neural Information Processing.

[18] Yang J., Nguyen M.N., San P.P., Li X., Krishnaswamy S. Deep Convolutional Neural Networks on Multichannel Time Series for Human Activity Recognition. Proceedings of the Twenty-Fourth International Joint Conference on Artificial Intelligence.

[19] Debache I., Jeantet L., Chevallier D., Bergouignan A., Sueur C. (2020). A Lean and Performant Hierarchical Model for Human Activity Recognition Using Body-Mounted Sensors. Sensors.

[20] Münzner S., Schmidt P., Reiss A., Hanselmann M., Stiefelhagen R., Dürichen R. (2017). CNN-Based Sensor Fusion Techniques for Multimodal Human Activity Recognition. Proceedings of the 2017 ACM International Symposium on Wearable Computers.

[21] Twomey N., Diethe T., Fafoutis X., Elsts A., McConville R., Flach P., Craddock I. (2018). A Comprehensive Study of Activity Recognition Using Accelerometers. Informatics.

[22] Ordonez F.J., Englebienne G., De Toledo P., Van Kasteren T., Sanchis A., Krose B. (2014). In-home activity recognition: Bayesian inference for hidden Markov models. IEEE Pervasive Comput..

[23] Zeng M., Nguyen L.T., Yu B., Ole J. M., Zhu J., Wu P., Zhang J. Convolutional neural networks for human activity recognition using mobile sensors. Proceedings of the 6th International Conference on Mobile Computing, Applications and Services.

[24] Hammerla N.Y., Halloran S., Ploetz T. (2016). Deep, convolutional, and recurrent models for human activity recognition using wearables. arXiv.

[25] ISO/IEC 19510:2013. https://www.iso.org/cms/render/live/en/sites/isoorg/contents/data/standard/06/26/62652.html.

[26] Moya Rueda F., Lüdtke S., Schröder M., Yordanova K., Kirste T., Fink G.A. Combining Symbolic Reasoning and Deep Learning for Human Activity Recognition. Proceedings of the 2019 IEEE International Conference on Pervasive Computing and Communications Workshops (PerCom Workshops).

[27] Dombrowski U., Riechel C., Schulze S. Enforcing Employees Participation in the Factory Planning Process. Proceedings of the 2011 IEEE International Symposium on Assembly and Manufacturing (ISAM).

[28] Nguyen M.N., Do N.H. (2016). Re-Engineering Assembly Line with Lean Techniques. Procedia CIRP.

[29] MbientLab—Wearable Bluetooth 9-Axis IMUs & Environmental Sensors. https://mbientlab.com/.

[30] Coren S. (1993). The Lateral Preference Inventory for Measurement of Handedness, Footedness, Eyedness, and Earedness: Norms for Young Adults. Bull. Psychon. Soc..

[31] De Kovel C.G.F., Carrión-Castillo A., Francks C. (2019). A Large-Scale Population Study of Early Life Factors Influencing Left-Handedness. Sci. Rep..

[32] Maurice P., Malaisé A., Amiot C., Paris N., Richard G.J., Rochel O., Ivaldi S. (2019). Human Movement and Ergonomics: An Industry-Oriented Dataset for Collaborative Robotics. Int. J. Robot. Res..

[33] Reining C., Rueda F.M., ten Hompel M., Fink G.A. Towards a Framework for Semi-Automated Annotation of Human Order Picking Activities Using Motion Capturing. Proceedings of the 2018 Federated Conference on Computer Science and Information Systems (FedCSIS).

[34] Nguyen L.T., Zeng M., Tague P., Zhang J. I Did Not Smoke 100 Cigarettes Today!: Avoiding False Positives in Real-World Activity Recognition. Proceedings of the 2015 ACM International Joint Conference on Pervasive and Ubiquitous Computing, ACM, UbiComp: 15.

[35] Perry J. (1992). Gait Analysis: Normal and Pathological Function. J. Pediatr. Orthop..

[36] Bokranz R., Landau K. (2012). Handbuch Industrial Engineering: Produktivitätsmanagement mit MTM. Band 1: Konzept.

[37] Whittle M.W. (2007). Gait Analysis: An Introduction.

[38] Reining C., Moya Rueda F., Niemann F., Fink G.A., ten Hompel M. Annotation Performance for Multi-Channel Time Series HAR Dataset in Logistics. Proceedings of the IEEE International Conference on Pervasive Computing and Communications (PerCom 2020).

[39] Moya Rueda F., Altermann E. (2020). Annotation Tool LARa. https://github.com/wilfer9008/Annotation_Tool_LARa.

[40] Kitchenham B., Brereton P. (2013). A systematic review of systematic review process research in software engineering. Inf. Softw. Technol..

[41] Kitchenham B., Pearl Brereton O., Budgen D., Turner M., Bailey J., Linkman S. (2009). Systematic literature reviews in software engineering—A systematic literature review. Inf. Softw. Technol..

[42] Kitchenham B. (2004). Procedures for Performing Systematic Reviews.

[43] Chen L., Zhao X., Tang O., Price L., Zhang S., Zhu W. (2017). Supply chain collaboration for sustainability: A literature review and future research agenda. Int. J. Prod. Econ..

[44] Caspersen C.J., Powell K.E., Christenson G.M. (1985). Physical activity, exercise, and physical fitness: Definitions and distinctions for health-related research. Public Health Rep..

[45] Vanrie J., Verfaillie K. Action Database. http://ppw.kuleuven.be/english/research/lep/resources/action.

[46] Theodoridis T. UCI Machine Learning Repository: Vicon Physical Action Data Set Data Set. http://archive.ics.uci.edu/ml/datasets/Vicon+Physical+Action+Data+Set.

[47] Shoaib M., Bosch S., Incel O.D., Scholten H., Havinga P.J.M. Research | Datasets | Pervasive Systems Group|University of Twente. http://www.utwente.nl/en/eemcs/ps/research/dataset/.

[48] Mandery C., Terlemez O., Do M., Vahrenkamp N., Asfour T. KIT Whole-Body Human Motion Database. http://motion-database.humanoids.kit.edu/.

[49] Jafari R., Chen C., Kehtarnavaz N. UTD Multimodal Human Action Dataset (UTD-MHAD). http://personal.utdallas.edu/~kehtar/UTD-MHAD.html.

[50] Kasebzadeh P., Hendeby G., Fritsche C., Gunnarsson F., Gustafsson F. IMU Dataset for Motion and Device Mode Classification. Proceedings of the 2017 International Conference on Indoor Positioning and Indoor Navigation (IPIN).

[51] Sztyler T. Human Activity Recognition. http://sensor.informatik.uni-mannheim.de/#dataset_dailylog.

[52] Vaizman Y., Ellis K., Lanckriet G. The ExtraSensory Dataset. http://extrasensory.ucsd.edu/.

[53] Chen C., Lu C.X., Markham A., Trigoni N. Dataset and Methods for Deep Inertial Odometry. http://deepio.cs.ox.ac.uk/.

[54] Müller M., Röder T., Clausen M., Krüger B., Weber A., Eberhardt B. Motion Database HDM05. http://resources.mpi-inf.mpg.de/HDM05/.

[55] Lustrek M., Kaluza B., Piltaver R., Krivec J., Vidulin V. UCI Machine Learning Repository: Localization Data for Person Activity Data Set. http://archive.ics.uci.edu/ml/datasets/Localization+Data+for+Person+Activity.

[56] Ugulino W., Cardador D., Vega K., Velloso E., Milidiú R., Fuks H. Human Activity Recognition. http://groupware.les.inf.puc-rio.br/har#ixzz2PyRdbAfA.

[57] Ahmed D.B. DLR—Institut Für Kommunikation Und Navigation—Data Set. http://www.dlr.de/kn/desktopdefault.aspx/tabid-12705/22182_read-50785/.

[58] Reiss A., Indlekofer I., Schmidt P., Van Laerhoven K. UCI Machine Learning Repository: PPG-DaLiA Data Set. http://archive.ics.uci.edu/ml/datasets/PPG-DaLiA.

[59] De la Torre F., Hodgins J., Montano J., Valcarcel S., Macey J., Forcada R. Quality of Life Grand Challenge | Kitchen Capture. http://kitchen.cs.cmu.edu/.

[60] Stisen A., Blunck H. UCI Machine Learning Repository: Heterogeneity Activity Recognition Data Set. http://archive.ics.uci.edu/ml/datasets/heterogeneity+activity+recognition.

[61] Vilarinho T., Bajer D.G., Dahl O.H., Egge I., Hegdal S.S., Lønes A., Slettevold J.N., Weggersen S.M. SINTEF-SIT/Project_gravity. http://github.com/SINTEF-SIT/project_gravity.

[62] Faye S., Louveton N., Jafarnejad S., Kryvchenko R., Engel T. An Open Dataset for Human Activity Analysis. http://kaggle.com/sasanj/human-activity-smart-devices.

[63] Sztyler T. Human Activity Recognition. http://sensor.informatik.uni-mannheim.de/#dataset_firstvision.

[64] Mohammed S., Gomaa W. HAD-AW Data-Set Benchmark For Human Activity Recognition Using Apple Watch. http://www.researchgate.net/publication/324136132_HAD-AW_Data-set_Benchmark_For_Human_Activity_Recognition_Using_Apple_Watch.

[65] Mandery C., Terlemez O., Do M., Vahrenkamp N., Asfour T. The KIT Whole-Body Human Motion Database. Proceedings of the 2015 International Conference on Advanced Robotics (ICAR).

[66] Vakanski A., Jun H.P., Paul D.R., Baker R.T. UI—PRMD. http://webpages.uidaho.edu/ui-prmd/.

[67] Sztyler T., Baur H. On-Body Localization of Wearable Devices: An Investigation of Position-Aware Activity Recognition. http://publications.wim.uni-mannheim.de/informatik/lski/Sztyler2016Localization.pdf.

[68] Vicon—Nexus. https://docs.vicon.com/display/Nexus26/Full+body+modeling+with+Plug-in+Gait.

[69] Roggen D., Plotnik M., Hausdorff J. UCI Machine Learning Repository: Daphnet Freezing of Gait Data Set. http://archive.ics.uci.edu/ml/datasets/Daphnet+Freezing+of+Gait.

[70] Maurice P., Malaisé A., Ivaldi S., Rochel O., Amiot C., Paris N., Richard G.J., Fritzsche L. AndyData-Lab-onePerson. http://zenodo.org/record/3254403#.XmDpQahKguV.

[71] Zhang W., Liu Z., Zhou L., Leung H., Chan A.B. Martial Arts, Dancing and Sports Dataset | VISAL. http://visal.cs.cityu.edu.hk/research/mads/.

[72] Trumble M., Gilbert A., Malleson C., Hilton A., Collomosse J. Total Capture: 3D Human Pose Estimation Fusing Video and Inertial Sensors. http://cvssp.org/data/totalcapture/.

[73] ANVIL: The Video Annotation Research Tool. http://www.anvil-software.org/.

[74] Bulling A., Blanke U., Schiele B. Andreas-Bulling/ActRecTut. http://github.com/andreas-bulling/ActRecTut.

[75] Sztyler T. Human Activity Recognition. http://sensor.informatik.uni-mannheim.de/#dataset_realworld.

[76] Zhang M., Sawchuk A.A. Human Activities Dataset. http://sipi.usc.edu/had/.

[77] Figshare. https://figshare.com/.

[78] UCI Machine Learning Repository. https://archive.ics.uci.edu/ml/index.php.

[79] Zenodo. https://zenodo.org/.

[80] GitHub. https://github.com.

[81] Dropbox. https://www.dropbox.com/.

[82] ResearchGate. https://www.researchgate.net/.

[83] Roggen D., Zappi P. Wiki:Dataset [Human Activity/Context Recognition Datasets]. http://har-dataset.org/doku.php?id=wiki:dataset.

[84] Carnegie Mellon University—CMU Graphics Lab - Motion Capture Library. http://mocap.cs.cmu.edu/.

[85] Vanrie J., Verfaillie K. (2004). Perception of Biological Motion: A Stimulus Set of Human Point-Light Actions. Behav. Res. Methods Instrum. Comput..

[86] Müller M., Röder T., Clausen M., Eberhardt B., Krüger B., Weber A.G. Documentation Mocap Database HDM05. https://www.researchgate.net/publication/231521391_Documentation_Mocap_database_HDM05.

[87] Yang A.Y., Giani A., Giannatonio R., Gilani K., Iyengar S., Kuryloski P., Seto E., Seppa V.P., Wang C., Shia V. D-WAR: Distributed Wearable Action Recognition. http://people.eecs.berkeley.edu/~yang/software/WAR/.

[88] Yang A.Y., Iyengar S., Kuryloski P., Jafari R. Distributed Segmentation and Classification of Human Actions Using a Wearable Motion Sensor Network. Proceedings of the 2008 IEEE Computer Society Conference on Computer Vision and Pattern Recognition Workshops.

[89] Forster K., Roggen D., Troster G. Unsupervised Classifier Self-Calibration through Repeated Context Occurences: Is There Robustness against Sensor Displacement to Gain?. Proceedings of the 2009 International Symposium on Wearable Computers.

[90] Spriggs E., De La Torre F., Hebert M. Temporal Segmentation and Activity Classification from First-Person Sensing. Proceedings of the 2009 IEEE Computer Society Conference on Computer Vision and Pattern Recognition Workshops.

[91] Sigal L., Balan A.O., Black M.J. HumanEva Dataset. http://humaneva.is.tue.mpg.de/datasets_human_1.

[92] Sigal L., Balan A.O., Black M.J. (2010). HumanEva: Synchronized Video and Motion Capture Dataset and Baseline Algorithm for Evaluation of Articulated Human Motion. Int. J. Comput. Vis..

[93] Sigal L., Balan A.O., Black M.J. HumanEva Dataset. http://humaneva.is.tue.mpg.de/datasets_human_2.

[94] Kaluža B., Mirchevska V., Dovgan E., Luštrek M., Gams M., de Ruyter B., Wichert R., Keyson D.V., Markopoulos P., Streitz N., Divitini M., Georgantas N., Mana Gomez A. (2010). An Agent-Based Approach to Care in Independent Living. Ambient Intelligence.

[95] Essid S., Lin X., Gowing M., Kordelas G., Aksay A., Kelly P., Fillon T., Zhang Q., Dielmann A., Kitanovski V. 3DLife ACM MM Grand Challenge 2011—Realistic Interaction in Online Virtual Environments. http://perso.telecom-paristech.fr/essid/3dlife-gc-11/.

[96] Essid S., Lin X., Gowing M., Kordelas G., Aksay A., Kelly P., Fillon T., Zhang Q., Dielmann A., Kitanovski V. (2013). A Multi-Modal Dance Corpus for Research into Interaction between Humans in Virtual Environments. J. Multimodal User Interfaces.

[97] McCall C., Reddy K., Shah M. CRCV | Center for Research in Computer Vision at the University of Central Florida. http://www.crcv.ucf.edu/data/UCF-iPhone.php.

[98] McCall C., Reddy K., Shah M. Macro-Class Selection for Hierarchical k-Nn Classification of Inertial Sensor Data. https://www.crcv.ucf.edu/papers/PECCS_2012.pdf.

[99] Theodoridis T., Hu H. Action Classification of 3D Human Models Using Dynamic ANNs for Mobile Robot Surveillance. Proceedings of the 2007 IEEE International Conference on Robotics and Biomimetics (ROBIO).

[100] Lockhart J.W., Weiss G.M., Xue J.C., Gallagher S.T., Grosner A.B., Pulickal T.T. WISDM Lab: Dataset. http://www.cis.fordham.edu/wisdm/dataset.php.

[101] Kwapisz J.R., Weiss G.M., Moore S.A. (2011). Activity Recognition Using Cell Phone Accelerometers. ACM SigKDD Explor. Newsl..

[102] Reyes-Ortiz J.L., Anguita D., Ghio A., Oneto L., Parra X. UCI Machine Learning Repository: Human Activity Recognition Using Smartphones Data Set. http://archive.ics.uci.edu/ml/datasets/human+activity+recognition+using+smartphones.

[103] Anguita D., Oneto L., Parra X., Reyes-Ortiz J.L. A Public Domain Dataset for Human Activity Recognition Using Smartphones. https://www.elen.ucl.ac.be/Proceedings/esann/esannpdf/es2013-84.pdf.

[104] Roggen D., Calatroni A., Rossi M., Holleczek T., Forster K., Troster G., Lukowicz P., Bannach D., Pirkl G., Ferscha A. Collecting Complex Activity Datasets in Highly Rich Networked Sensor Environments. Proceedings of the 2010 Seventh International Conference on Networked Sensing Systems (INSS).

[105] Reiss A., Stricker D. Introducing a New Benchmarked Dataset for Activity Monitoring. Proceedings of the 2012 16th International Symposium on Wearable Computers.

[106] Zhang M., Sawchuk A.A. USC-HAD: A Daily Activity Dataset for Ubiquitous Activity Recognition Using Wearable Sensors. Proceedings of the 2012 ACM Conference on Ubiquitous Computing.

[107] Kwapisz J.R., Weiss G.M., Moore S.A. WISDM Lab: Dataset. http://www.cis.fordham.edu/wisdm/dataset.php.

[108] Lockhart J.W., Weiss G.M., Xue J.C., Gallagher S.T., Grosner A.B., Pulickal T.T. Design Considerations for the WISDM Smart Phone-Based Sensor Mining Architecture. Proceedings of the Fifth International Workshop on Knowledge Discovery from Sensor Data.

[109] Barshan B. UCI Machine Learning Repository: Daily and Sports Activities Data Set. http://archive.ics.uci.edu/ml/datasets/Daily+and+Sports+Activities.

[110] Barshan B., Yuksek M.C. (2014). Recognizing Daily and Sports Activities in Two Open Source Machine Learning Environments Using Body-Worn Sensor Units. Comput. J..

[111] Bachlin M., Plotnik M., Roggen D., Maidan I., Hausdorff J., Giladi N., Troster G. (2009). Wearable Assistant for Parkinson’s Disease Patients With the Freezing of Gait Symptom. IEEE Trans. Inf. Technol. Biomed..

[112] Shoaib M., Scholten H., Havinga P. Towards Physical Activity Recognition Using Smartphone Sensors. Proceedings of the 2013 IEEE 10th International Conference on Ubiquitous Intelligence and Computing and 2013 IEEE 10th International Conference on Autonomic and Trusted Computing.

[113] Medrano C., Igual R., Plaza I., Castro M. Fall ADL Data | EduQTech. http://eduqtech.unizar.es/en/fall-adl-data/.

[114] Medrano C., Igual R., Plaza I., Castro M. (2014). Detecting Falls as Novelties in Acceleration Patterns Acquired with Smartphones. PLoS ONE.

[115] Ugulino W., Cardador D., Vega K., Velloso E., Milidiú R., Fuks H., Barros L.N., Finger M., Pozo A.T., Gimenénez-Lugo G.A., Castilho M. (2012). Wearable Computing: Accelerometers’ Data Classification of Body Postures and Movements. Advances in Artificial Intelligence—SBIA 2012.

[116] Casale P., Pujol O., Radeva P. UCI Machine Learning Repository: Activity Recognition from Single Chest-Mounted Accelerometer Data Set. http://archive.ics.uci.edu/ml/datasets/Activity+Recognition+from+Single+Chest-Mounted+Accelerometer.

[117] Casale P., Pujol O., Radeva P. (2012). Personalization and User Verification in Wearable Systems Using Biometric Walking Patterns. Pers. Ubiquitous Comput..

[118] Banos O., Toth M.A., Amft O. UCI Machine Learning Repository: REALDISP Activity Recognition Dataset Data Set. http://archive.ics.uci.edu/ml/datasets/REALDISP+Activity+Recognition+Dataset.

[119] Banos O., Toth M., Damas M., Pomares H., Rojas I. (2014). Dealing with the Effects of Sensor Displacement in Wearable Activity Recognition. Sensors.

[120] Shoaib M., Bosch S., Incel O., Scholten H., Havinga P. (2014). Fusion of Smartphone Motion Sensors for Physical Activity Recognition. Sensors.

[121] Casale P. UCI Machine Learning Repository: User Identification From Walking Activity Data Set. http://archive.ics.uci.edu/ml/datasets/User+Identification+From+Walking+Activity.

[122] Shoaib M., Bosch S., Incel O., Scholten H., Havinga P. (2016). Complex Human Activity Recognition Using Smartphone and Wrist-Worn Motion Sensors. Sensors.

[123] Stisen A., Blunck H., Bhattacharya S., Prentow T.S., Kjærgaard M.B., Dey A., Sonne T., Jensen M.M. Smart Devices Are Different: Assessing and MitigatingMobile Sensing Heterogeneities for Activity Recognition. Proceedings of the 13th ACM Conference on Embedded Networked Sensor Systems.

[124] Ahmed D.B., Frank K., Heirich O. Recognition of Professional Activities with Displaceable Sensors. Proceedings of the 2015 IEEE 82nd Vehicular Technology Conference (VTC2015-Fall).

[125] Wojtusch J., von Stryk O. HuMoD Database Human Motion Dynamics on Actuation Level. https://www.sim.informatik.tu-darmstadt.de/res/ds/humod/.

[126] Wojtusch J., von Stryk O. HuMoD—A Versatile and Open Database for the Investigation, Modeling and Simulation of Human Motion Dynamics on Actuation Level. Proceedings of the 2015 IEEE-RAS 15th International Conference on Humanoid Robots (Humanoids).

[127] Vilarinho T., Farshchian B., Bajer D.G., Dahl O.H., Egge I., Hegdal S.S., Lones A., Slettevold J.N., Weggersen S.M. A Combined Smartphone and Smartwatch Fall Detection System. Proceedings of the 2015 IEEE International Conference on Computer and Information Technology; Ubiquitous Computing and Communications; Dependable, Autonomic and Secure Computing; Pervasive Intelligence and Computing.

[128] Zappi P., Lombriser C., Stiefmeier T., Farella E., Roggen D., Benini L., Tröster G., Verdone R. (2008). Activity Recognition from On-Body Sensors: Accuracy-Power Trade-Off by Dynamic Sensor Selection. Wireless Sensor Networks.

[129] Reyes-Ortiz J.L., Oneto L., Monsonís A.S., Parra X. UCI Machine Learning Repository: Smartphone-Based Recognition of Human Activities and Postural Transitions Data Set. http://archive.ics.uci.edu/ml/datasets/Smartphone-Based+Recognition+of+Human+Activities+and+Postural+Transitions.

[130] Reyes-Ortiz J.L., Oneto L., Samà A., Parra X., Anguita D. (2016). Transition-Aware Human Activity Recognition Using Smartphones. Neurocomputing.

[131] Chen C., Jafari R., Kehtarnavaz N. UTD-MHAD: A Multimodal Dataset for Human Action Recognition Utilizing a Depth Camera and a Wearable Inertial Sensor. Proceedings of the 2015 IEEE International Conference on Image Processing (ICIP).

[132] Palumbo F., Gallicchio C., Pucci R., Micheli A. UCI Machine Learning Repository: Activity Recognition System Based on Multisensor Data Fusion (AReM) Data Set. http://archive.ics.uci.edu/ml/datasets/Activity+Recognition+system+based+on+Multisensor+data+fusion+%28AReM%29.

[133] Palumbo F., Gallicchio C., Pucci R., Micheli A. (2016). Human Activity Recognition Using Multisensor Data Fusion Based on Reservoir Computing. J. Ambient Intell. Smart Environ..

[134] Sztyler T., Carmona J., Völker J., Stuckenschmidt H., Koutny M., Desel J., Kleijn J. (2016). Self-Tracking Reloaded: Applying Process Mining to Personalized Health Care from Labeled Sensor Data. Transactions on Petri Nets and Other Models of Concurrency XI.

[135] Vaizman Y., Ellis K., Lanckriet G. (2017). Recognizing Detailed Human Context in the Wild from Smartphones and Smartwatches. IEEE Pervasive Comput..

[136] Vögele A., Krüger B. HDM12 Dance - Documentation on a Data Base of Tango Motion Capture. http://cg.cs.uni-bonn.de/en/publications/paper-details/voegele-2016-HDM12/.

[137] Davis K.A., Owusu E.B. UCI Machine Learning Repository: Smartphone Dataset for Human Activity Recognition (HAR) in Ambient Assisted Living (AAL) Data Set. http://archive.ics.uci.edu/ml/datasets/Smartphone+Dataset+for+Human+Activity+Recognition+%28HAR%29+in+Ambient+Assisted+Living+%28AAL%29.

[138] Casilari E., A.Santoyo-Ramón J. UMAFall: Fall Detection Dataset (Universidad de Malaga). http://figshare.com/articles/UMA_ADL_FALL_Dataset_zip/4214283.

[139] Casilari E., Santoyo-Ramón J.A., Cano-García J.M. (2017). UMAFall: A Multisensor Dataset for the Research on Automatic Fall Detection. Procedia Comput. Sci..

[140] Faye S., Louveton N., Jafarnejad S., Kryvchenko R., Engel T. An Open Dataset for Human Activity Analysis Using Smart Devices. https://hal.archives-ouvertes.fr/hal-01586802.

[141] Kasebzadeh P., Hendeby G., Fritsche C., Gunnarsson F., Gustafsson F. Parinaz Kasebzadeh: Research. http://users.isy.liu.se/rt/parka23/research.html.

[142] Zhang W., Liu Z., Zhou L., Leung H., Chan A.B. (2017). Martial Arts, Dancing and Sports Dataset: A Challenging Stereo and Multi-View Dataset for 3D Human Pose Estimation. Image Vis. Comput..

[143] Vakanski A., Jun H.p., Paul D., Baker R. (2018). A Data Set of Human Body Movements for Physical Rehabilitation Exercises. Data.

[144] Sucerquia A., López J.D., Vargas-Bonilla J.F. SisFall | SISTEMIC. http://sistemic.udea.edu.co/en/investigacion/proyectos/english-falls/.

[145] Sucerquia A., López J., Vargas-Bonilla J. (2017). SisFall: A Fall and Movement Dataset. Sensors.

[146] Trumble M., Gilbert A., Malleson C., Hilton A., Collomosse J. (2017). Total Capture: 3D Human Pose Estimation Fusing Video and Inertial Sensors. Br. Mach. Vis. Assoc..

[147] Micucci D., Mobilio M., Napoletano P. UniMiB SHAR. http://www.sal.disco.unimib.it/technologies/unimib-shar/.

[148] Micucci D., Mobilio M., Napoletano P. (2017). UniMiB SHAR: A Dataset for Human Activity Recognition Using Acceleration Data from Smartphones. Appl. Sci..

[149] Martinez-Villaseñor L., Ponce H., Brieva J., Moya-Albor E., Núñez Martínez J., Peñafort Asturiano C. HAR-UP. http://sites.google.com/up.edu.mx/har-up/.

[150] Martínez-Villaseñor L., Ponce H., Brieva J., Moya-Albor E., Núñez Martínez J., Peñafort Asturiano C. (2019). UP-Fall Detection Dataset: A Multimodal Approach. Sensors.

[151] Ashry S., Elbasiony R., Gomaa W. (2018). An LSTM-Based Descriptor for Human Activities Recognition Using IMU Sensors.

[152] Chereshnev R., Kertész-Farkas A. Romanchereshnev/HuGaDB. http://github.com/romanchereshnev/HuGaDB.

[153] Chereshnev R., Kertész-Farkas A., van der Aalst W.M., Ignatov D.I., Khachay M., Kuznetsov S.O., Lempitsky V., Lomazova I.A., Loukachevitch N., Napoli A., Panchenko A., Pardalos P.M. (2018). HuGaDB: Human Gait Database for Activity Recognition from Wearable Inertial Sensor Networks. Analysis of Images, Social Networks and Texts.

[154] Chen C., Zhao P., Lu C.X., Wang W., Markham A., Trigoni N. OxIOD: The Dataset for Deep Inertial Odometry. https://www.researchgate.net/publication/327789960_OxIOD_The_Dataset_for_Deep_Inertial_Odometry.

[155] Turan A., Barshan B. UCI Machine Learning Repository: Simulated Falls and Daily Living Activities Data Set Data Set. http://archive.ics.uci.edu/ml/datasets/Simulated+Falls+and+Daily+Living+Activities+Data+Set.

[156] Özdemir A., Barshan B. (2014). Detecting Falls with Wearable Sensors Using Machine Learning Techniques. Sensors.

[157] Tits M., Laraba S., Caulier E., Tilmanne J., Dutoit T. UMONS-TAICHI. http://github.com/numediart/UMONS-TAICHI.

[158] Tits M., Laraba S., Caulier E., Tilmanne J., Dutoit T. (2018). UMONS-TAICHI: A Multimodal Motion Capture Dataset of Expertise in Taijiquan Gestures. Data Brief.

[159] Reiss A., Indlekofer I., Schmidt P., Van Laerhoven K. (2019). Deep PPG: Large-Scale Heart Rate Estimation with Convolutional Neural Networks. Sensors.

[160] Niemann F., Reining C., Moya Rueda F., Nair N.R., Steffens J.A., Fink G.A., ten Hompel M. (2020). Logistic Activity Recognition Challenge (LARa)—A Motion Capture and Inertial Measurement Dataset.

